# Changes in aorta hemodynamics in Left-Right Type 1 bicuspid aortic valve patients after replacement with bioprosthetic valves: An in-silico study

**DOI:** 10.1371/journal.pone.0301350

**Published:** 2024-04-16

**Authors:** Shantanu Bailoor, Jung-Hee Seo, Stefano Schena, Rajat Mittal

**Affiliations:** 1 Department of Mechanical Engineering, The Johns Hopkins University, Baltimore, MD, United States of America; 2 Division of Cardiothoracic Surgery, Medical College of Wisconsin, Milwaukee, WI, United States of America; Coventry University, UNITED KINGDOM

## Abstract

Bicuspid aortic valve (BAV) is the most common cardiac congenital abnormality with a high rate of concomitant aortic valve and ascending aorta (AAo) pathologic changes throughout the patient’s lifetime. The etiology of BAV-related aortopathy was historically believed to be genetic. However, recent studies theorize that adverse hemodynamics secondary to BAVs also contribute to aortopathy, but their precise role, specifically, that of wall shear stress (WSS) magnitude and directionality remains controversial. Moreover, the primary therapeutic option for BAV patients is aortic valve replacement (AVR), but the role of improved post-AVR hemodynamics on aortopathy progression is also not well-understood. To address these issues, this study employs a computational fluid dynamics model to simulate personalized AAo hemodynamics before and after TAVR for a small cohort of 6 Left-Right fused BAV patients. Regional distributions of five hemodynamic metrics, namely, time-averaged wall shear stress (TAWSS) and oscillating shear index (OSI), divergence of wall shear (DWSS), helicity flux integral & endothelial cell activation potential (ECAP), which are hypothesized to be associated with potential aortic injury are computed in the root, proximal and distal ascending aorta. BAVs are characterized by strong, eccentric jets, with peak velocities exceeding 4 m/s and axially circulating flow away from the jets. Such conditions result in focused WSS loading along jet attachment regions on the lumen boundary and weaker, oscillating WSS on other regions. The jet attachment regions also show alternating streaks of positive and negative DWSS, which may increase risk for local tissue stretching. Large WSS magnitudes, strong helical flows and circumferential WSS have been previously implicated in the progression of BAV aortopathy. Post-intervention hemodynamics exhibit weaker, less eccentric jets. Significant reductions are observed in flow helicity, TAWSS and DWSS in localized regions of the proximal AAo. On the other hand, OSI increases post-intervention and ECAP is observed to be low in both pre- and post-intervention scenarios, although significant increases are also observed in this ECAP. These results indicate a significant alleviation of pathological hemodynamics post AVR.

## 1. Introduction

**Fig 1:**
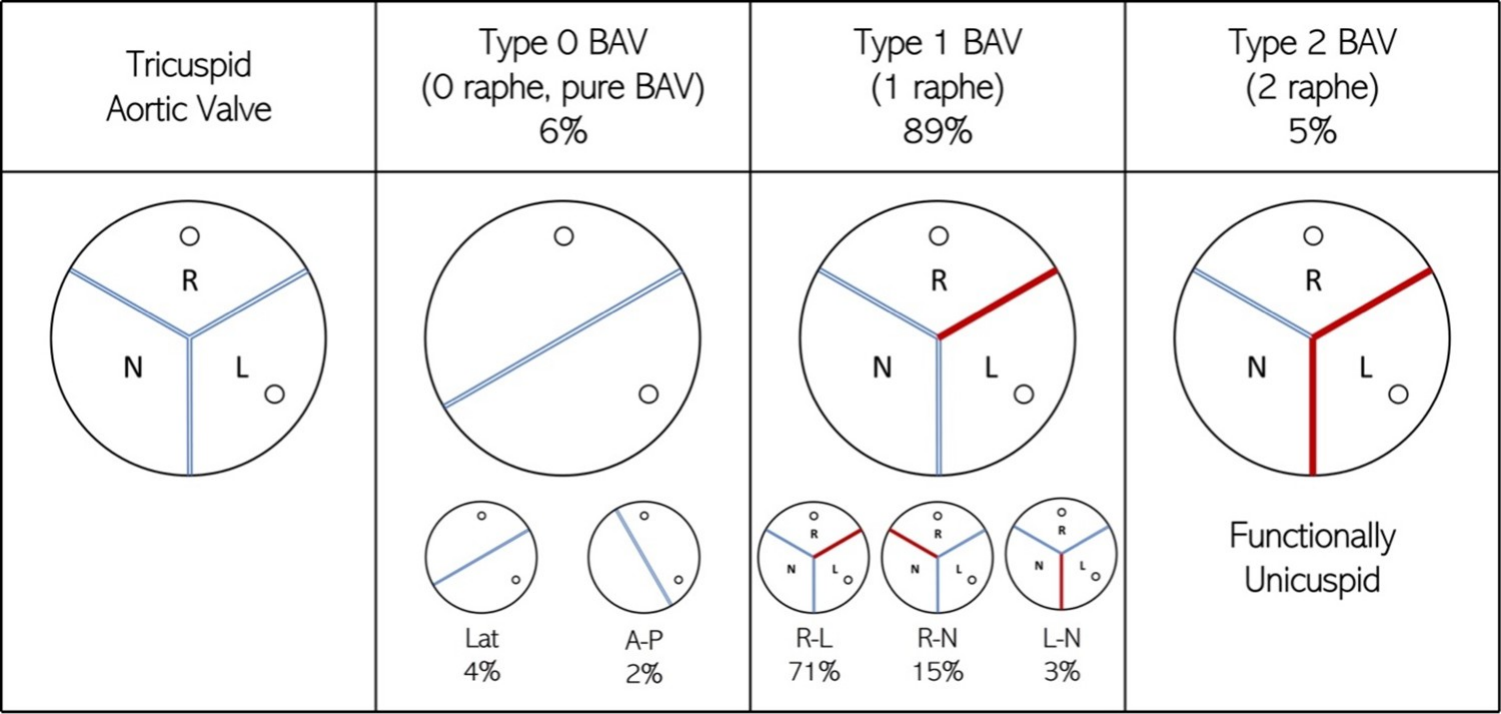
Morphological differences between tricuspid aortic valve and the different types of BAV, classified using the Sievers classification system [3]. The blue lines represent edges of mobile leaflets, while the red lines represent fused raphes. The left and right coronary cusps show circles representing coronary ostia. For each BAV morphotype, its prevalence in terms of percentage of the total BAV population is also specified.

Bicuspid aortic valve (BAV) is the most common cardiac congenital abnormality affecting approximately 2% of the general population [1], of which approximately 75% are male [2]. Children born with BAVs are typically asymptomatic, and they are either diagnosed incidentally through an echocardiogram performed for other reasons or go undiagnosed into adulthood when valvular leaflet degeneration eventually leads to clinically relevant symptoms. Bicuspid aortic valves usually present with either two full formed leaflets (pure BAV) or one free leaflet and two leaflets which are conjoined due to congenital underdevelopment leading to a malformed, fibrous, commissure, called raphe. Sievers and Schmidtke [3] proposed a classification for different BAV types based on the number of raphes present and the leaflets between which fusion exists. The different types of BAVs, according to this system, and their prevalence rates are illustrated in Fig 1. Of the different phenotypes, type-1 BAV is the most prevalent, making up nearly 90% of the BAV population. Among the non “pure” BAVs patient population, type-1 with a fusion of the right and left cusps with one raphe (R-L type-1), occur in more than 70% of cases.

Although BAV anatomy can often achieve normal valvular function, it is associated with a high rate of structural deterioration of the aortic valve (AV), such as calcific stenosis, insufficiency and is subject to higher risk of infective endocarditis. Similarly, the presence of BAV may affect the structure of the aortic root and the ascending aorta (AAo), in the form of concurrent coarctation, progressive dilation, aneurysmatic degeneration, intimal dissection or even rupture. These adverse outcomes are known to occur in around 40–60% of those with BAVs, putting them at a 6-9x risk for fatal complications compared to subjects with normal tricuspid valves [4]. While the role of genetic anomalies (“genetic theory”) in the fate of the AAo of BAV patients has been long acknowledged [2,5–10], Atkins et al. [11] recently hypothesized that adverse hemodynamics (“hemodynamic theory”) might also influence the pathogenesis and progression of BAV-related aortopathy. Della Corte et al. [12] found that regurgitant BAVs were strongly associated with root aneurysms, whereas AAo dilation was more prevalent with hypertensive blood flow and stenotic BAVs. The tubular aortopathy phenotype secondary to stenotic BAVs is commonly observed to begin at the sinotubular junction (STJ) and mainly involves the convexity of the AAo [13,14]. Furthermore, the type of aortopathy resulting from BAV also appears to depend on the BAV morphotype. Indeed, root dilation was predicted by the presence of R-L BAVs without significant stenosis and R-N BAVs with severe regurgitation, whereas AAo dilation was more prevalent in stenotic R-L BAVs and those without regurgitation [12].

Thus, altered hemodynamics specific to BAV morphotype appear to be important determinants of the risk and type of aortopathy, but the underlying mechanism is not yet clearly understood. Particularly, the role of wall shear stress (WSS) magnitude and directionality in aortopathy risk remains a topic of debate. Historically, low and oscillating shear stress and higher residence times secondary to flow stagnation have been strongly correlated with intraluminal thrombus (ILT) formation and consequently risk aneurysm development in the common iliac artery [15] and abdominal aorta [16,17]. Likewise, several studies have found a strong association between highly bidirectional WSS of relatively low magnitude and the risk of cerebral aneurysm growth and rupture [18–20]. On the other hand, chronically high WSS is also known to alter endothelial response which stimulates outward vascular remodeling, causing an expansion of the arterial diameter [21–23]. This adaptation is an effort to downregulate abnormally high WSS to physiological levels, and vessel expansion has been observed to halt when WSS returns to baseline levels, irrespective of the flow-rate [23–25]. Focused investigations into the association between BAV aortopathy and the underlying blood flow appear to suggest that the hemodynamic determinants of aortic diameter modifications might follow a different mechanism. A recent study by Dux-Santoy et al. [26] compared WSS on the AAo in 46 BAV patients to 44 healthy volunteers using time-resolved 3D MRI and found that regions exposed to low and oscillating wall shear stress in BAV patients did not match those with the highest prevalence of dilation. In other studies, the group also showed, through cardiac MRI analysis and in silico modeling that BAV patients exhibited strong helical flows in the aorta, with increased axial circulation [27] and circumferential wall shear stress [28] compared to healthy controls. Further, they showed that the magnitude and circumferential component of the local WSS vector correlated strongly in BAV patients with the risk of AAo growth rate [29]. In another study [30], the group employed several machine learning algorithms to establish biomarkers which most strongly correlate with BAV-disease and found that jet velocity angle consistently predicted BAV hemodynamics, along with forward/ reverse velocity and indicators of rotational flow, such as helicity, vorticity and circulation. 4D flow MRI analysis of 60 subjects by Minderhoud et al. [31] showed that larger WSS angle, and consequently, larger circumferential WSS component, were strong predictors of dilation in BAV patients.

Another controversial issue around BAV aortopathy relates to changes in the rate of disease progression post-AV replacement (AVR). A severely restricted BAV, regardless of the etiology, is currently treated by replacing the diseased valve, but the fate of the aorta may evolve toward progression or stabilization of the disease, even after relief of the valve obstruction. Naito et al. [32] observed that aortas of BAV patients continue to exhibit severe histological lesions in regions in direct contact with eccentric jets post AVR. The authors concluded that hemodynamic factors may continue to contribute to late progression of BAV aortopathy post AVR. Hattori et al. [33] found, using post AVR CT-scans of 14 BAV patients, that AAo diameters continued to grow, on average, at a rate of 1.02 mm/year. On the other hand, Charitos et al. [34] found that BAV patients with normal aortic root hemodynamics after AVR exhibited similar rates of AAo growth over time as those observed among the general population. Girdauskas and Borger [35] also emphasize the role of post-intervention hemodynamics in aortopathy progression, but note that the tubular AAo phenotype follows a benign prognosis post AVR. Regeer et al. [36] found through analysis of 93 AVR candidates with BAV that aortic dilation rates reduced from 0.42 mm/year to 0.28 mm/year postoperatively. Thus, there is consensus that AAo hemodynamics are linked to stenotic BAV aortopathy risk and post AVR progression, but their precise role remains unclear.

Based on these premises, it would be useful to understand the degree to which the AAo hemodynamics and turbulence, as well as the associated AAo WSS profile is altered in BAV patients after the implantation of a prosthetic valve. In addition, the capability of predicting ahead of the procedure, the post-implant AAo hemodynamics for these patients could assist cardiac surgeons in making a better assessment in terms of AAo management both at the time of AVR and during follow-up. In the current study, we utilize image-based in-silico computational models to compare the presurgical AAo hemodynamics as well as wall-shear metrics that are correlated with aortopathy, with the corresponding metrics after a virtual implantation of a bioprosthetic trileaflet aortic valve in the same BAV patient. We focus here on bioprosthetic valves since these are increasingly the choice for aortic valve replacement [37]. We note that several previous numerical investigations have described AAo hemodynamic characteristics secondary to BAVs in detail, including jet displacement, jet angle, flow helicity and the associated asymmetric, and oscillating wall loading [38–41]. However, these studies either assumed idealized aorta or BAV anatomy [38,39], or ignored dynamic leaflet motion [41], or are limited by the choice of BAV phenotype [40] and turbulence model [39]. We overcame previous limitations in the current literature and based our study on a small cohort of severely stenotic BAV-patients (N = 7) for whom AVR was performed with a bioprosthetic trileaflet aortic valve (BTAV). Direct numerical simulations (DNS) are performed on models based on pre-intervention CT scans obtained for each patient with a BAV as well a corresponding hypothetical post-intervention case using a TAV virtually implanted in place of the BAV. To avoid confounding effects, the same ventricular function, including heart rate, stroke volume and velocity profile, as well as root & AAo anatomy are used in both cases. AAo hemodynamics and several WSS-derived metrics are evaluated over the cohort with BAV and TAV phenotypes, and regional differences in WSS magnitude and directionality are evaluated to identify the nature of abnormal WSS loading on high-risk sections of the AAo. This approach is advantageous in the following two ways: (i) DNS ensure that turbulent flow features are resolved, and consequently, that WSS is accurately estimated, and (ii) employing the same root + AAo anatomy in the BAV and TAV cases eliminates the effect of any AAo morphological differences associated with severely stenotic BAVs on the flow fields. This also facilitates isolating the effect of a change in valve phenotype on risk and progression of aortopathy. The results of the present study describe AAo hemodynamics and WSS distributions on the lumen boundary, secondary to severely stenotic BAVs and changes in these hemodynamic metrics post-AVR. Comparing differences between the pre- and post-intervention WSS magnitude and direction and its related quantities can help understand the underlying hemodynamic contributors to BAV-related aortopathy and its possible resolution following AVR.

## 2. Methods

### 2.1. Fluid-structure interaction valve dynamics solver

In large blood vessels such as the aorta, blood flow can be assumed to be homogeneous and Newtonian with density ρ (= 1,060 kg/m^3^) dynamic viscosity μ (= 4.0 cP) [42]. Thus, blood flow is modeled by the three-dimensional, incompressible Navier-Stokes’ equations, which are discretized using a second-order accurate central-difference scheme, and integrated on three-dimensional, non-uniform Cartesian grids. Such a grid topology simplifies the discretization of partial derivatives, boundary condition enforcement at domain boundaries and parallelization of the code. All immersed surfaces are represented by three-dimensional surface meshes with triangular elements. The interaction between the fluid and structural subsystems is modeled using the sharp interface immersed boundary method of Mittal et al. [43]. The solver has previously been successfully employed for direct numerical simulations of flow through stenosed aortic valves (AS) [44,45], mitral valves[46] and left ventricles [46,47], as well as several other biological flow systems [48,49]. The solver is coupled with a versatile reduced degree-of-freedom (rDOF) valve model [45,50–54] which considerably simplifies the structural subsystem, reduces simulation time can be adapted to patient-specific annular morphology and accurately simulate various valvular pathologies. The model is used to simulate the response of severely stenotic BAVs and healthy TAVs to physiological blood flow for a set of patient-specific AAo models. The governing equations of the rDOF model, its adaptation to patient-specific anatomy and its ability to mimic several valve conditions are described in detail in S1 Appendix and S2 Appendix.

### 2.2. Patient inclusion/exclusion criteria

Patients undergoing AVR (N = 6) were enrolled in this study and screened to meet the following inclusion criteria:

1. All enrolled patients presented with severe bicuspid aortic valve stenosis (AOA < 1 cm^2^, mean gradient ≥40 mm Hg, peak velocity ≥4.0 m/sec).
2. Intermediate to high surgical risk interpreted as STS-PROM score >4%, or low surgical risk (STS-PROM score <4%).

Additionally, the following criteria were used to exclude patients from the study:

1. Concurrent severe coronary artery disease.
2. Previous cardiac surgery.
3. Low flow/low gradient aortic valve stenosis.

Chronic renal insufficiency, defined as glomerular filtration rate (GFR) < 60.

History of congenital aortopathies (i.e. Marfan, Loeys-Dietz syndrome, etc) or other concurrent collagen disease.

The study protocol was approved by the Johns Hopkins Medical Institute Institutional Review Board (IRB) with IRB number: IRB00177900. All patient data (CT scan, echocardiographic images and reports) were obtained after a written informed consent and were anonymized before use. The types of BAV observed in the patient cohort are listed for each patient in Table 1. The CT images for patient 1 may be used to replicate the results described herein and are provided by Mittal [55].

**Table 1:** A description of the patient cohort describing the Sievers type of bicuspid valve observed in each patient (RL-T1: R-L Type 1; AP-T0: A-P Type 0).

Patient	1	2	3	4	5	6
Sievers Type	RL-T1	RL-T1	RL-T1	RL-T1	RL-T1	RL-T1

**Fig 2:**
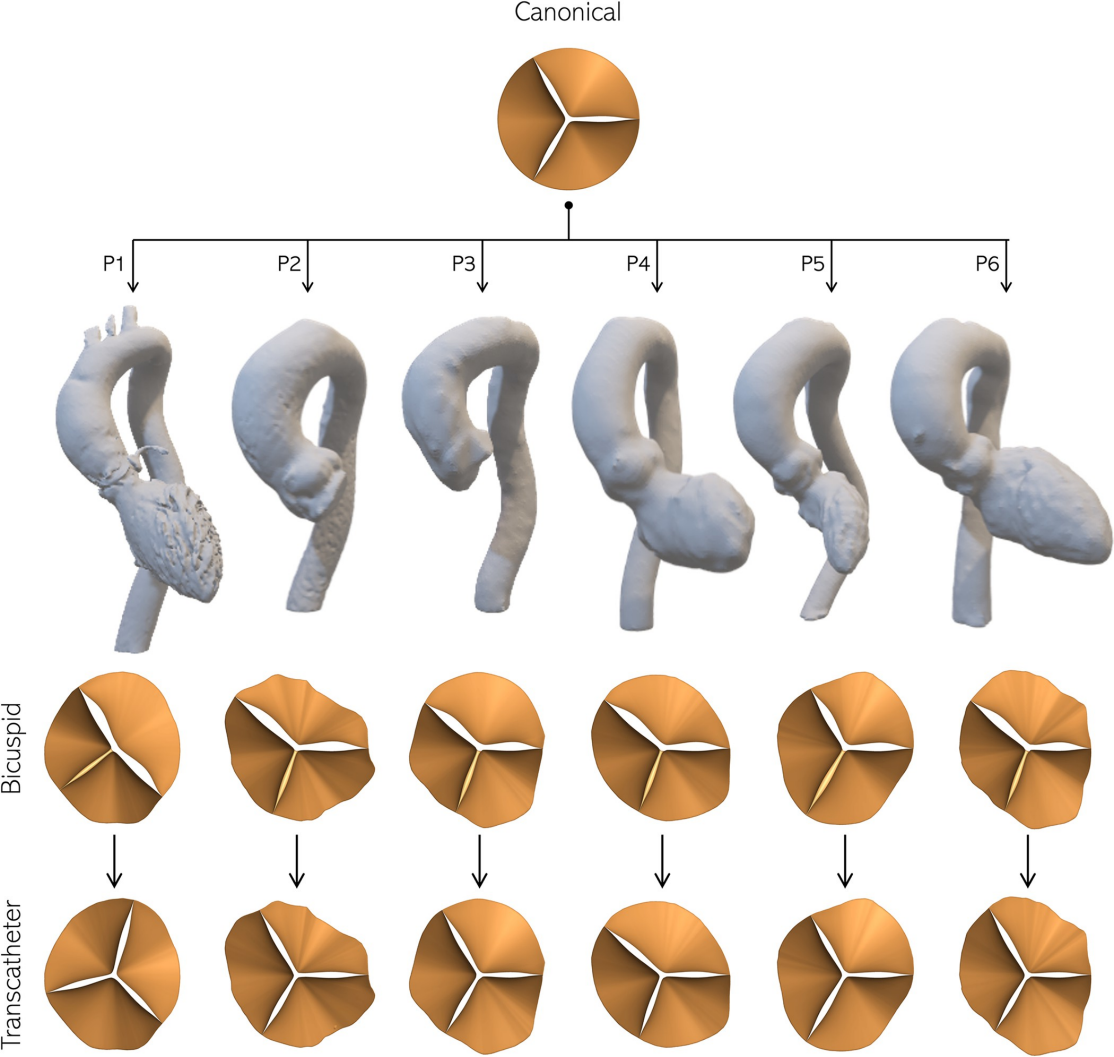
Adaptation of the canonical AV model to the annular morphology of the 7 patients enrolled in the study, shown using 3D models of their respective aorta CT-scans. The first row of patient-specific valves shows native BAVs, with leaflets fitted to individual sinus cusps. The second row shows the corresponding hypothetical prosthetic TAVs fitted to the aortic annulus with the three leaflets equal in angular span.

Of the recruited patients, the first six presented with the left-right (LR) fused BAV with raphe (Sievers Type-1), while the seventh had a Sievers Type-0 BAV. Accordingly, the rDOF valve model described in section 2.1 is adapted to the seven patient anatomies as shown in Fig 2. The same annular shape is used to compute the BAV and TAV shapes. Type-1 BAVs are modeled by fusing the left and right leaflets of a tricuspid valve using a virtual raphe, while the type-0 BAV is modeled by starting with two leaflets and stretching them in the circumferential direction. The BAV leaflets are azimuthally adjusted to fit those of the sinus cusps, so that the commissure lines between the two leaflets were aligned with the interface between the appropriate cusps. On the other hand, the tricuspid valve shapes are assumed to be derived from prosthetic valves, each spanning 120°, conforming to the patient’s annular boundary.

### 2.3. Dynamic response tuning

Once the model is set to obtain desired leaflet shapes and kinematic features, its dynamic response can be tuned by determining leaflet inertia and stiffness in terms of *α* & κ. In a previous study [51], we simulated flow inside a canonical aorta morphology using the idealized valve model and showed that under identical inflow conditions with *α* = 40*kg*/*m*^2^, *κ* = 10,600 *Pa*/*m*, leaflet motion, quantified as the instantaneous projected valve open area (PVOA), was in good agreement with that predicted by the 3D non-linear finite-element valve model of de Tullio and Pascazio [56]. Thus, for the rest of this study, healthy leaflets are modeled using these values for *α* & *κ*. As previously stated, aortic stenosis via leaflet thickening can be modeled by increasing either *α* or *κ* over its respective baseline value.

### 2.4. Simulation setup

**Fig 3:**
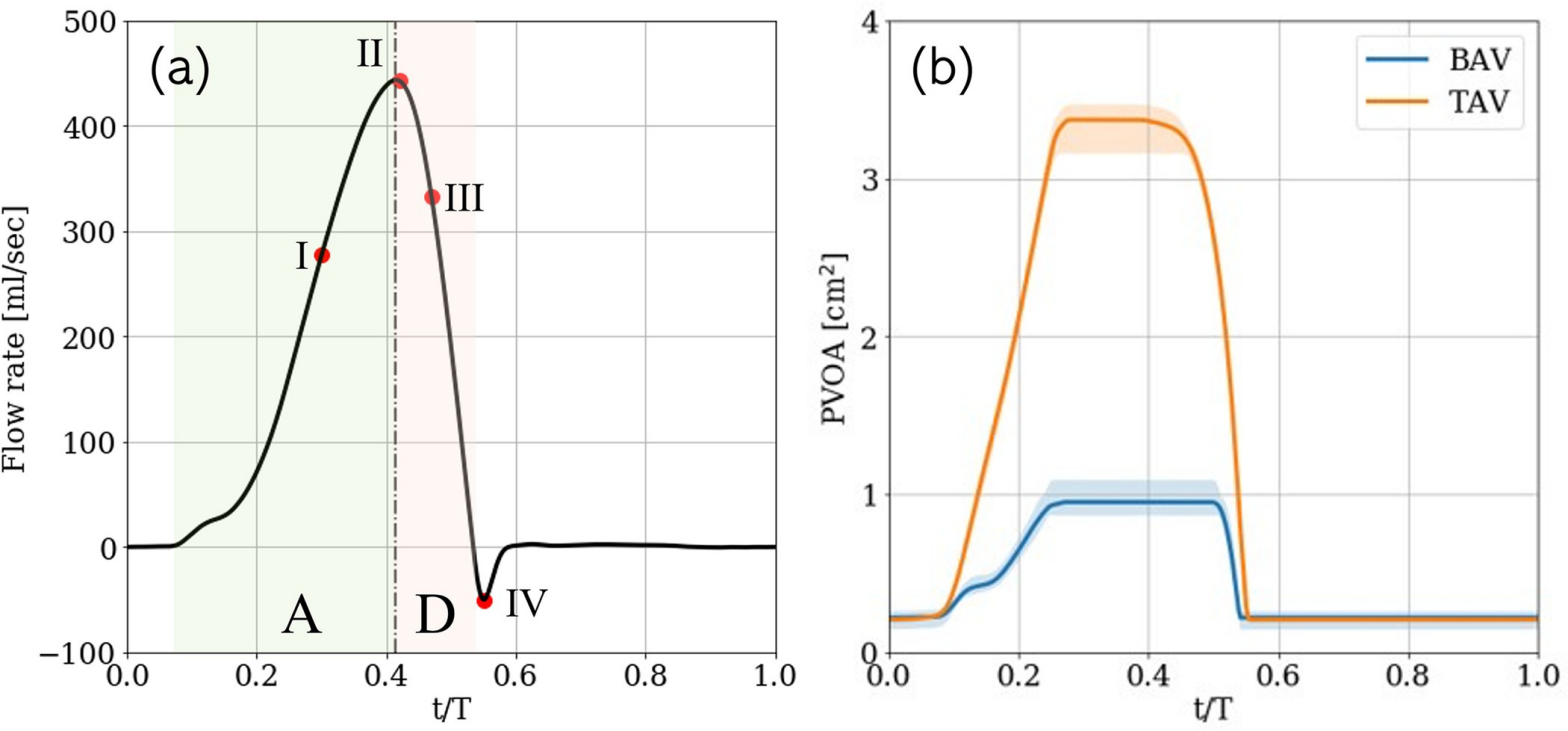
(a) Flow-rate profile used to drive simulations with each patient anatomy, marked with (A) acceleration and (D) deceleration phases, and four analysis instants at t/T = 0.30, 0.42, 0.47 & 0.55 and (b) evolution of resulting projected valve open area (PVOA) obtained using BAV and TAV over the dataset. The solid lines represent the respective PVOAs averaged over all patients, while the shaded regions indicate the corresponding time-varying bounds (min/max) on PVOA.

The aorta models described in section 2.2 are truncated at the LVOT and the aortic arch and extensions are created at each truncated end in local the axial direction. Information regarding time-varying flow-rate profiles were not available for all patients. Hence, an inflow-profile is used which is synthesized from a pulse duplicator system employed for *in vitro* experiments of transvalvular flow (Fig 3(A); courtesy L.P. Dasi, Cardiovascular Fluid Mechanics Lab, Georgia Institute of Technology). It is characterized by a heart rate of 60 bpm, systolic ejection period of approximately 440 msec, and stroke volume of approximately 92 ml. It comprises of a gradual acceleration (A) phase (duration ≈340 ms), followed by rapid deceleration (D) (duration ≈100 ms), with a peak at around *t*/*T* = 0.42. The simulation Reynolds number, based on peak systolic inflow velocity (*U*_peak_) and annular area (*A*_LVOT_), assuming blood viscosity to be 4.0 cP, is $\left(Re_{\mathrm{peak}}=\frac{U_{\mathrm{peak}}}{\nu }\sqrt{\frac{4A_{\mathrm{LVOT}}}{\pi }}\right)$ 6400. Each simulation was run on 256 CPUs for approximately 48 hours, thus requiring approximately 12,000 CPU hours each.

Simulations are performed for each patient anatomy using both valve (BAV and BTAV) phenotypes and the flow-rate profile described above. For each patient, several hemodynamic metrics are evaluated which have been proposed to predict possible vascular endothelial damage for the pre- (BAV) and post-intervention (TAV) cases. Correlations between changes in different metrics and zones at risk for aortopathy are used to investigate the role of WSS in the progression of aortic dilation and/ or dissection. For ease of comparison between patients, all aorta models are scaled so that the LVOT cross-sectional area is the same as that of a 23 mm diameter valve. The time-varying projected valve open area (PVOA) for the simulated BAV and TAV cases in the dataset are shown in Fig 3(B). The fused BAV leaflets are assumed to be rigidly held while the mobility of any non-fused leaflets can be varied to achieve desired orifice areas. The resulting peak PVOA averaged across the patient cohort were 0.951 cm^2^ & 3.372 cm^2^, for the BAVs and TAVs respectively. Each case is simulated using a uniform, Cartesian grid with grid spacing of 0.5 mm. This grid resolution was determined through the grid-refinement study described in S3 Appendix. This grid resolution and tuned leaflet parameters are also shown to result in good agreement with clinical patient-specific echocardiographic measurements, as shown in S4 Appendix.

### 2.5. Assessed hemodynamic quantities

**Fig 4:**
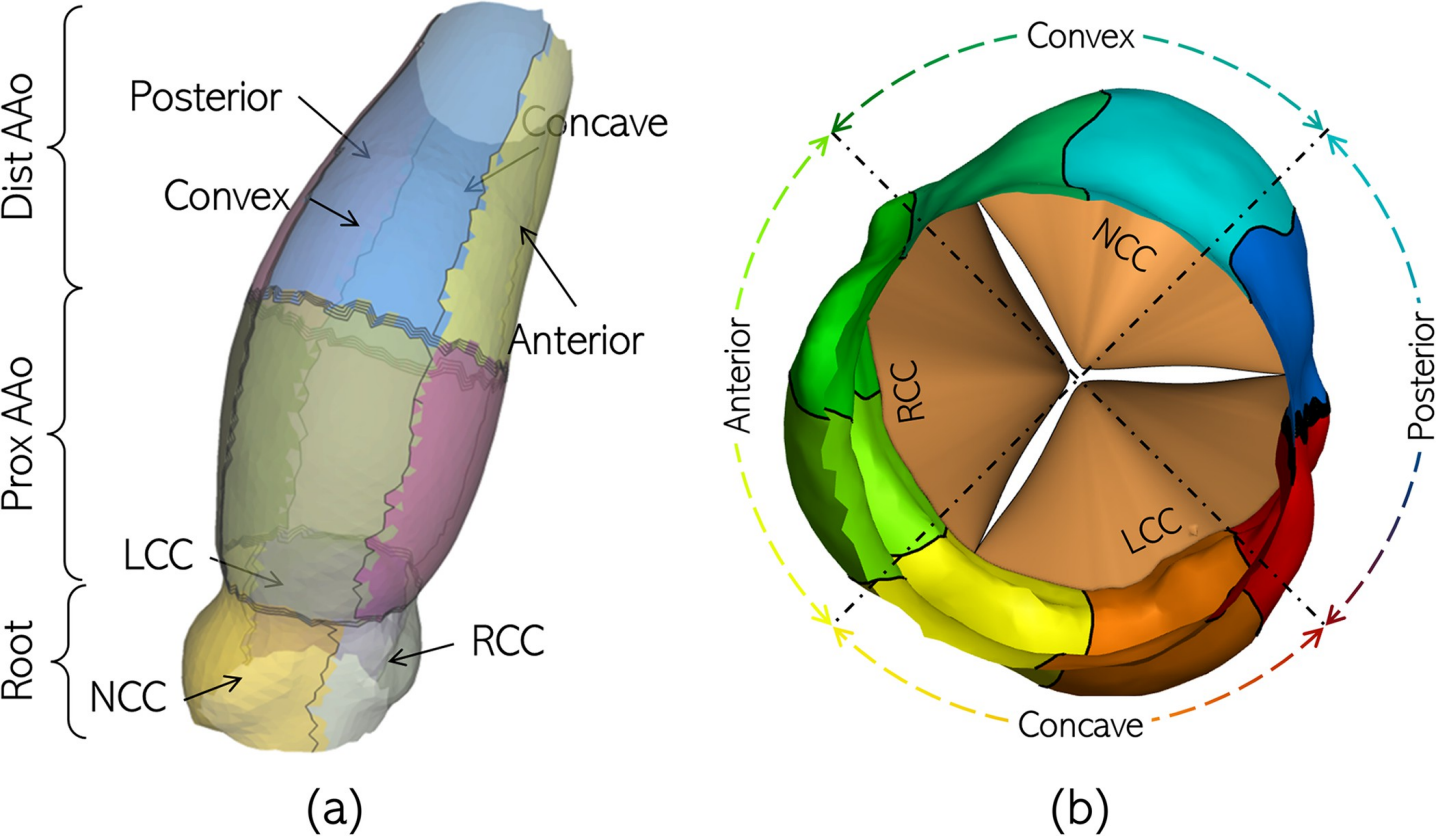
Illustration of the 11 wall shear analysis zones for patient 1’s aorta anatomy.

For the purpose of analysis, the aorta lumen boundary is divided into three axial regions corresponding to the aortic root, proximal AAo (PAAo) and distal AAo (DAAo). The boundary between the PAAo and DAAo is approximately halfway between the sino-tubular junction and the aortic arch. The root is further divided into the non- (NCC), right- (RCC) and left-coronary cusps (LCC), while the PAAo and DAAo are divided into their respective convex, anterior, concave and posterior surfaces. In this manner, the aorta is divided into 11 analysis zones, which are illustrated in Fig 4 for Patient 1.

The following flow and WSS-derived quantities are evaluated to analyze the pre- and post-intervention hemodynamics and assess the associated risk of aortopathy:

#### 2.5.1. Helicity flux integral

Flow helicity in the AAo can also be quantified in terms of the helicity flux (H) through a section, defined as shown in Eq (1)

$$H=\int_{S}\left[\nabla \times ((\vec{u}\cdot \hat{t})\hat{t})\right]\cdot \hat{n}dS\;\begin{array}{c} H>0;\;\mathrm{ccw}\;\mathrm{rotation}\\ H<0;\;\mathrm{cw}\;\mathrm{rotation} \end{array}$$ (1)

The helicity flux through an axial section plane has been previously used by Cao et al. [39] to quantify circulating flow in the AAo with BAVs, and may be viewed as the surface integral of the out-of-plane vorticity. The sign of the integral refers to the direction of in-plane rotation using the right-hand rule: positive integrals correspond to counter-clockwise bulk rotation, while negative integrals indicate clockwise rotation. Alternatively, using Kelvin’s circulation theorem, it may be interpreted as the line integral of the of circulation contained by the section boundary. Using Helmholtz’s theorem, it may also be interpreted as the area integral of the out-of-plane (axial) vorticity.

#### 2.5.2. Time-averaged wall shear stress magnitude

The time-averaged wall shear stress magnitude is calculated as shown in Eq (2), where $\vec{\tau_{w}}$ represents the local WSS vector:

$$\bar{\left|\vec{\tau_{w}}\right|}=\frac{1}{T}\int_{0}^{T}|\vec{\tau_{w}}|dt$$ (2)

#### 2.5.3. Oscillating shear index

The Oscillating Shear Index (OSI) is a measure of the bidirectionality of the local WSS vector over a defined time interval and is calculated as shown in Eq (3). It is a scalar integral quantity which takes values in the range [0,0.5], such that it is 0 for a WSS field which does not reverse direction at any instant during the period of integration, and 0.5 for a purely oscillating WSS field.


$$\mathrm{OSI}=\frac{1}{2}\left[1-\frac{\left|\int_{0}^{T}\vec{\tau_{w}}dt\right|}{\int_{0}^{T}|\vec{\tau_{w}}|dt}\right]$$ (3)

#### 2.5.4. Divergence of wall shear stress

**Fig 5:**
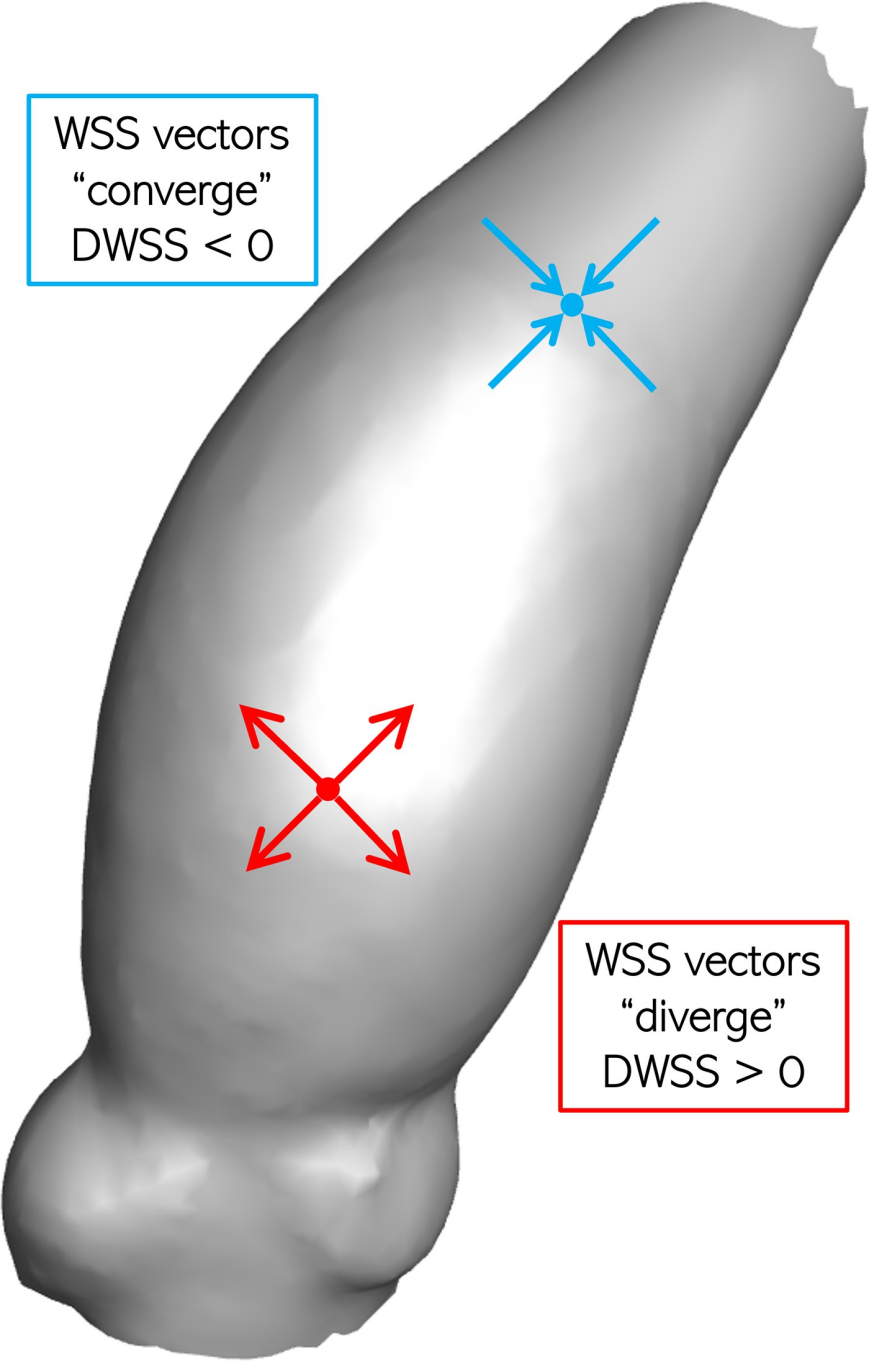
Schematic showing diverging (red) and converging (blue) WSS vectors, leading to positive and negative DWSS, respectively.

The next analyzed metric is the divergence of wall shear vector (DWSS), which is a scalar-valued measure indicating whether the surface distribution of WSS results in a local stretching or a compression effect on the lumen boundary. Mathematically, the local DWSS is calculated as shown in Eq (4) and has been previously proposed to assess the risk of intracranial aneurysm rupture [57]. Fig 5 illustrates a schematic showing two generic locations on the AAo lumen boundary, each experiencing a different loading condition. The first location (red) experiences positive DWSS, such that local WSS vectors point outwards (diverge) and as a result, forces act to tangentially stretch the lumen boundary. Diverging vectors on the lumen boundary could also result from material transport from the lumen towards the boundary. This could imply an elevated risk of pro-inflammatory material deposition on the aorta wall and, consequently, increased endothelial cell inflammation and damage. On the other hand, the other location (blue) experiences negative DWSS such that WSS vectors locally point inwards and, consequently, tangentially compressive forces act on the lumen boundary. Convergent WSS vectors could promote material transport away from the boundary, towards the lumen.


$$\mathrm{DWSS}=\nabla \cdot \vec{\tau_{W}}$$ (4)

#### 2.5.5. Endothelial cell activation potential

The final metric assessed is the endothelial cell activation potential (ECAP), calculated as shown in Eq (5). This metric has been previously proposed to predict the risk for aneurysm formation in major arteries such as the abdominal aorta [17]. The authors proposed a critical value of **1.4 Pa**^**‒1**^, over which the risk of intraluminal thrombus formation was high.


$$\mathrm{ECAP}=\frac{\mathrm{OSI}}{\mathrm{TAWSS}}$$ (5)

From Eq (5) it may be inferred that high ECAP regions coincide with those experiencing low and oscillating WSS.

## 3. Results

### 3.1. Ascending aorta hemodynamics

**Fig 6:**
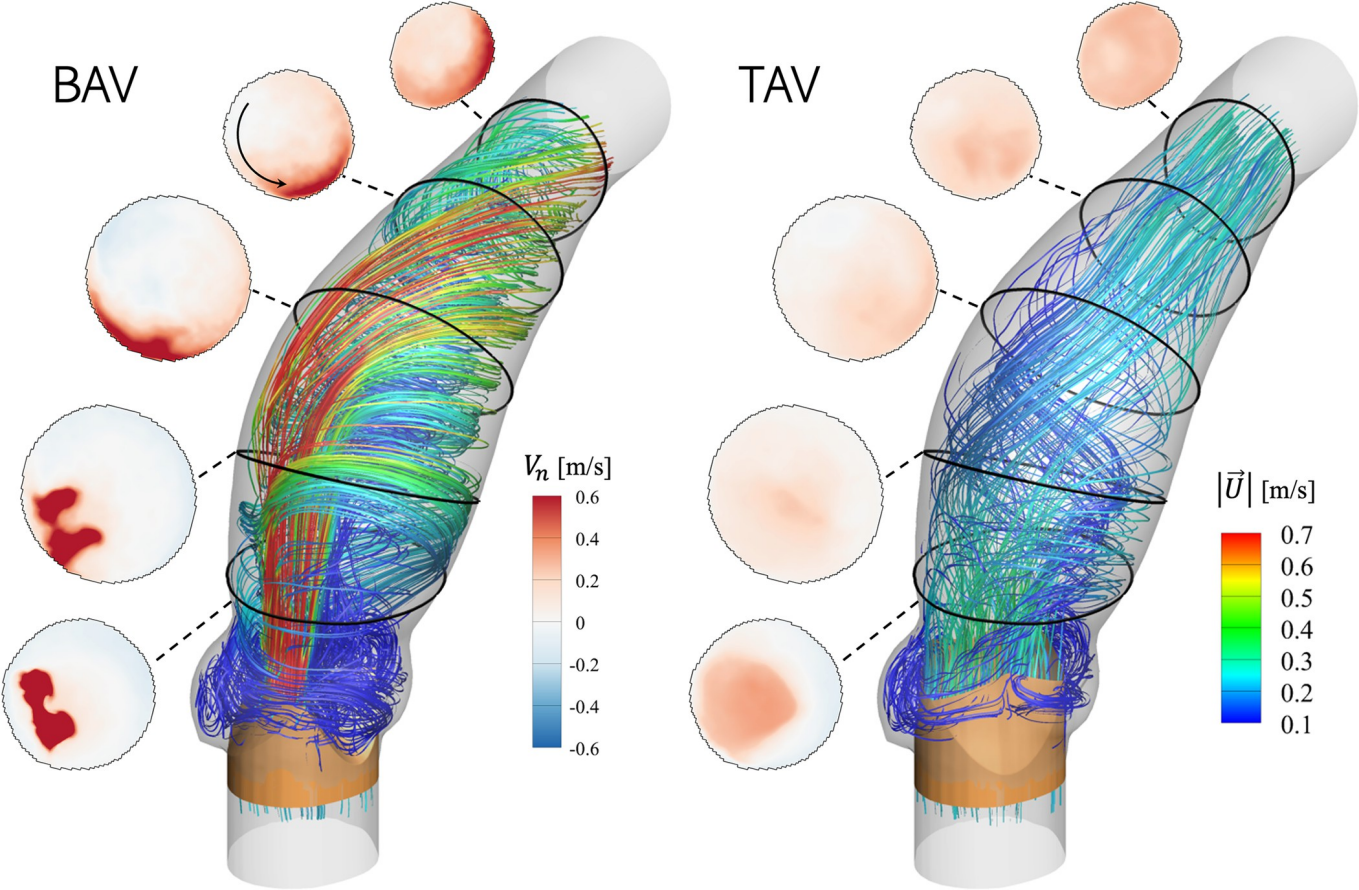
Flow in the AAo, visualized using streamlines of time-averaged velocity vectors and colored using average velocity magnitude $\left|\vec{U}\right|$ for (left) BAV and (right) BTAV using Patient 1’s anatomy. Additionally, in each figure, axial velocity (*V*_*n*_) contours at 5 analysis sections are shown.

In this section, differences in flow-fields observed between AAos with BAV and TAV are described. For the sake of convenience, simulations with BAVs and TAVs are also referred to as “pre-intervention” and “post-intervention” cases, respectively. Differences in flow-fields influence the biomechanical stresses on the aorta lumen boundary, thus, affecting the risk of aortopathy. Flow visualization for Patient 1 are presented in detail to describe hemodynamic differences in the two scenarios. Later in the section, the distributions of several hemodynamic metrics, averaged over the patient cohort in the pre- and post-intervention cases, are described over important analysis zones to identify regions at greater risk for vascular damage and those which might experience improved hemodynamics after valve replacement. Fig 6 shows the time-averaged flow fields in Patient 1’s AAo in the (left) pre- (BAV) and (right) post-intervention (TAV) cases, visualized using streamlines colored by velocity magnitude $\left|\vec{U}\right|$. Additionally, five analysis sections are defined along the aorta axis, starting at the sino-tubular junction (STJ) and moving towards the aortic arch. These sections, shown in Fig 6(A), are used to illustrate the evolution of the aortic jet, visualized using contours of the axial (or normal) component of velocity *V*_*n*_. The cycle-averaged pre-intervention aortic jet velocity magnitude is ≈1.1 m/sec, which reduces to ≈0.35 m/sec with a TAV due to an increase in the valve orifice area. The asymmetric stenosis in a type-1 BAV results in a narrow jet which is skewed toward the convex surface of the aorta lumen boundary. As the jet propagates downstream, it appears to rotate towards the anterior surface, as it bends along the AAo curvature. This jet rotation is also evidenced by its shape and location, relative to the five analysis sections; first, it attaches to the convex wall of the PAAo (sections 1, 2). As it propagates downstream (sections 3, 4), it forms a crescent shape, with most of its momentum concentrated close to the wall. At the same time, circulating flow patterns with counter-clockwise rotation (looking upstream towards the AV) and a weak retrograde motion (<0.4 m/sec) are observed to originate near the concave surface of the PAAo and extend towards the concave and posterior surfaces of the DAAo. This secondary helical flow which sets up on the opposite side of the wall causes the jet to rotate towards the anterior surface as it propagates in the DAAo. The strong attached jet on one side of the aorta wall, together with the weak helical flow on the other results in regionally asymmetric loading which may increase the susceptibility to vascular damage. In the post-intervention case, the larger PVOA results in lower jet velocity, because of mass conservation. The time-averaged jet velocity near the STJ (section 1) is observed to be ≈0.2 m/sec. The jet appears to be more circular and less eccentric, compared to the pre-intervention case. As it propagates through the aorta, the jet expands, and its momentum diffuses in the radial direction. In sections 2–5, the jet is observed to occupy the entire cross-section, evidenced by the presence of antegrade flow (red). The streamlines also appear to be aligned with the aorta axis throughout the AAo, except for a short, dilated section of the PAAo, where the flow exhibits weak helicity. The most notable difference in the post-intervention flow visualization is the absence of regionally asymmetric flow distribution and the reduced jet velocity.

**Fig 7:**
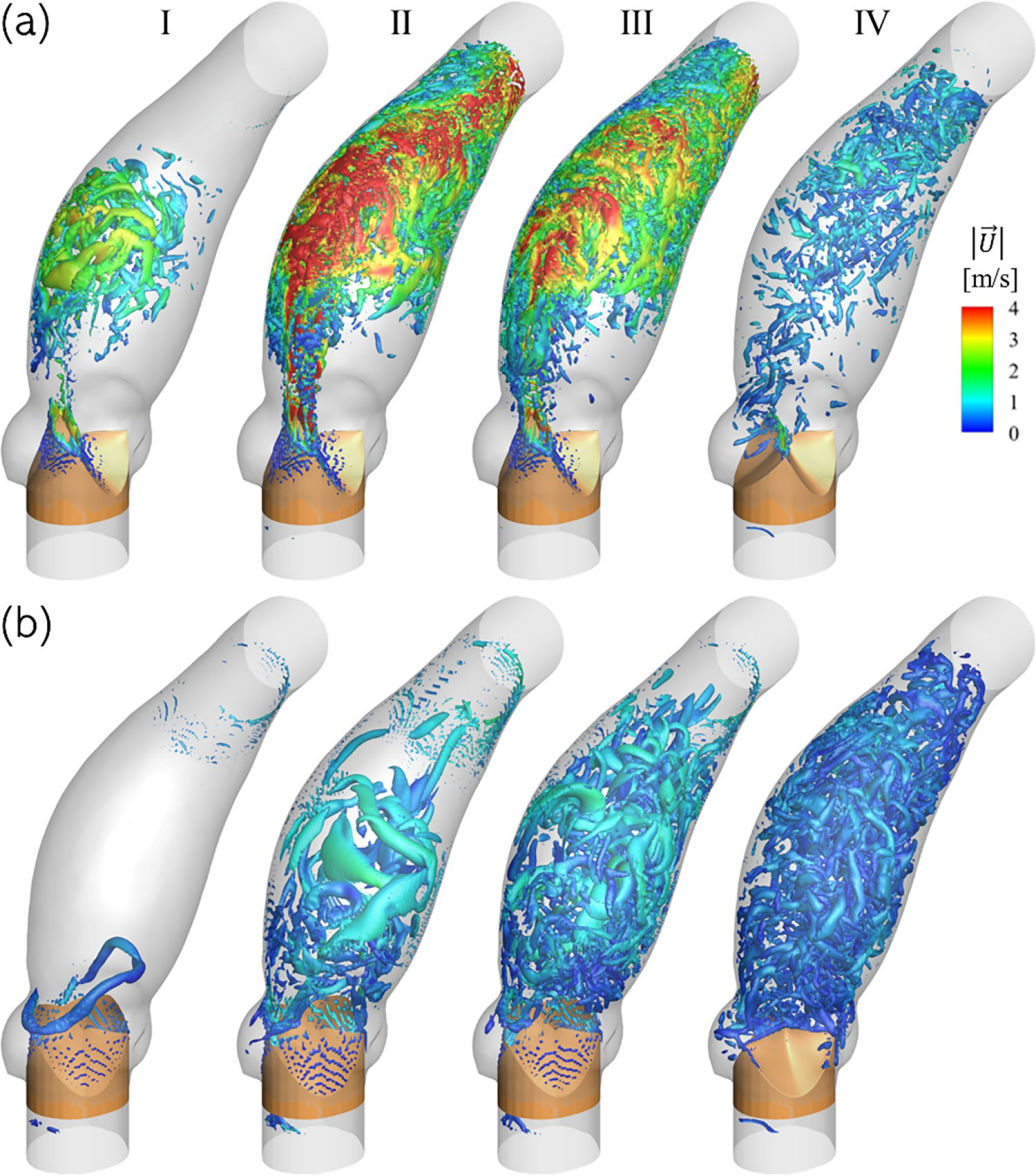
Comparison of vortex structures, visualized using isosurfaces of Q-criterion and colored using contours of velocity magnitude in the (top) pre-intervention and (bottom) post-intervention cases for Patient 1 anatomy at the four analysis time-instants described in Fig 3(A). Due to the large difference in velocity magnitude in the two cases and for ease of visualization, the pre- and post-intervention isosurfaces are plotted for *Q* = 10^5^ sec^‒2^ & *Q* = 2×10^4^ sec^‒2^, respectively.

Fig 7 illustrates vortex structures visualized using isosurfaces of Q-criterion in the pre- and post-intervention cases, at the four instants marked in Fig 3(A). The pre-intervention case shows isosurfaces of *Q* = 10^5^ sec^‒2^, wherein major vortex structures first appear during the acceleration phase (I) where the jet contacts the convex surface of the aorta wall. Around peak systole (II) the jet velocity is observed to increase past 8.0 m/sec and the aortic jet, observed using the high velocity contours, appears to tilt towards the anterior surface as it bends along the aorta wall curvature. At the same time, vortex structures appear near the aorta concave and posterior surfaces, moving with relatively lower velocity (≈2‒3 m/sec) than the jet. These surfaces are not in direct contact with the aortic jet, and the nearby flow represents secondary helical flow which sets up insde the AAo due to shear effects of the strong jet and centripetal acceleration due to fluid motion in a curved vessel. As the flow decelerates (III) the jet velocity magnitude decreases, evidenced by fewer streaks of Q-criterion with $|\vec{U}|>4.0$ m/sec, but the helical flow pattern continues and extends further upstream towards the valve. Finally, after systolic ejection has ended and the valve has closed completely (IV), only some residual, low velocity (≈1.0 m/sec) helical motion remains, which eventually dissipates during diastole.

Due to the larger PVOA associated with the post-intervention (TAV) case, the flow is characterized by significantly lower velocities and, consequently, velocity gradients throughout the cardiac cycle. Therefore, the *Q*-criterion isosurfaces used to visualize the post-intervention vortex structures in Fig 7(B) are of weaker strength (*Q* = 2×10^4^ sec^‒2^) compared to those used in the pre-intervention case. An initial vortex ring is (I) formed at the leading end of the aortic jet which convects downstream as the jet through the aorta. The high Reynolds number jet has a shear layer where more instabilities grow, resulting in larger, but weaker vortex structures in the proximal AAo around peak systole (II). The maximum peak systolic flow velocity is 1.59 m/sec. During deceleration (III), the adverse pressure gradient further facilitates instability growth, due to which, more vortex structures are formed throughout the AAo. After systolic ejection ends (IV), only slow, axially circulating flow in the AAo remains with velocity <1.0 m/sec, which decays towards quiescence by the end of the cardiac cycle. Thus, valve replacement helps to reduce peak jet velocities to physiological levels and reduces turbulent vortical structures, as evidenced by a weaker *Q*-criterion field.

**Fig 8:**
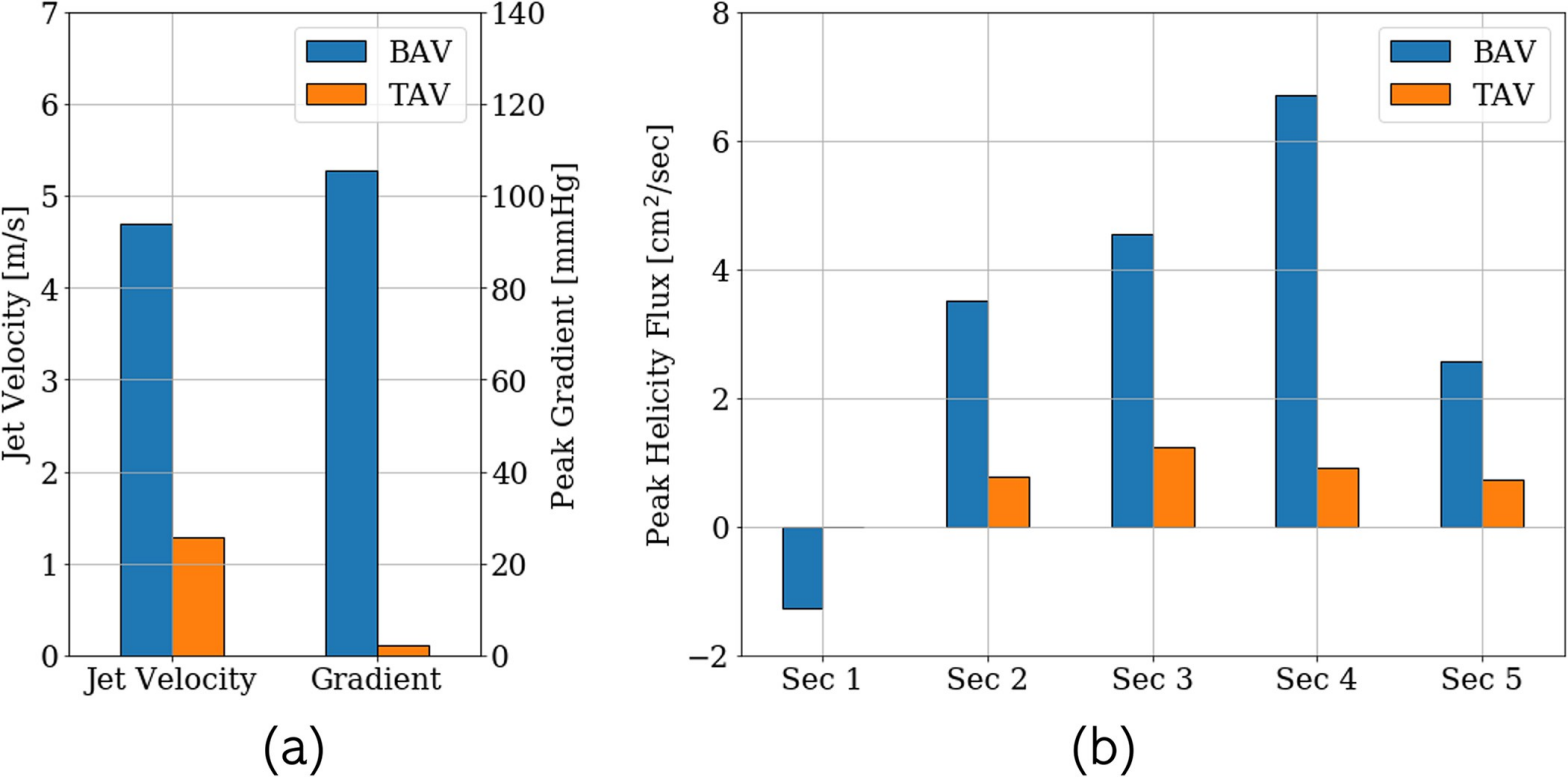
Comparison of the (a) peak systolic aortic jet velocity and transvalvular gradients, and (b) peak helicity flux in the 5 analysis sections defined in Fig 6 averaged over the patient cohort in the (blue) pre- and (orange) post-intervention cases.

Fig 8(A) shows a comparison of the cohort-averaged peak systolic aortic jet velocity and transvalvular gradient between the pre- and post-intervention cases. The severely stenotic BAVs were associated with peak systolic jet velocities of about 4.7 m/sec. Post intervention, the jet peak jet velocity is observed to be 1.27 m/sec. Likewise, the peak transvalvular gradients calculated as the difference between the peak systolic pressures measured immediately upstream and downstream from the aortic valve, reduced from 105 mm Hg to 2.24 mm Hg (<5 mm Hg). Helicity flux is evaluated for the 5 analysis sections defined in Fig 6, and a cohort-wide comparison between their respective pre- and post-intervention values is presented in Fig 8(B). Both these changes indicate a post-intervention resolution of adverse hemodynamic conditions which can prevent further damage to valvular, vascular as well as ventricular health.

### 3.2. Analysis of hemodynamic wall loading

**Fig 9:**
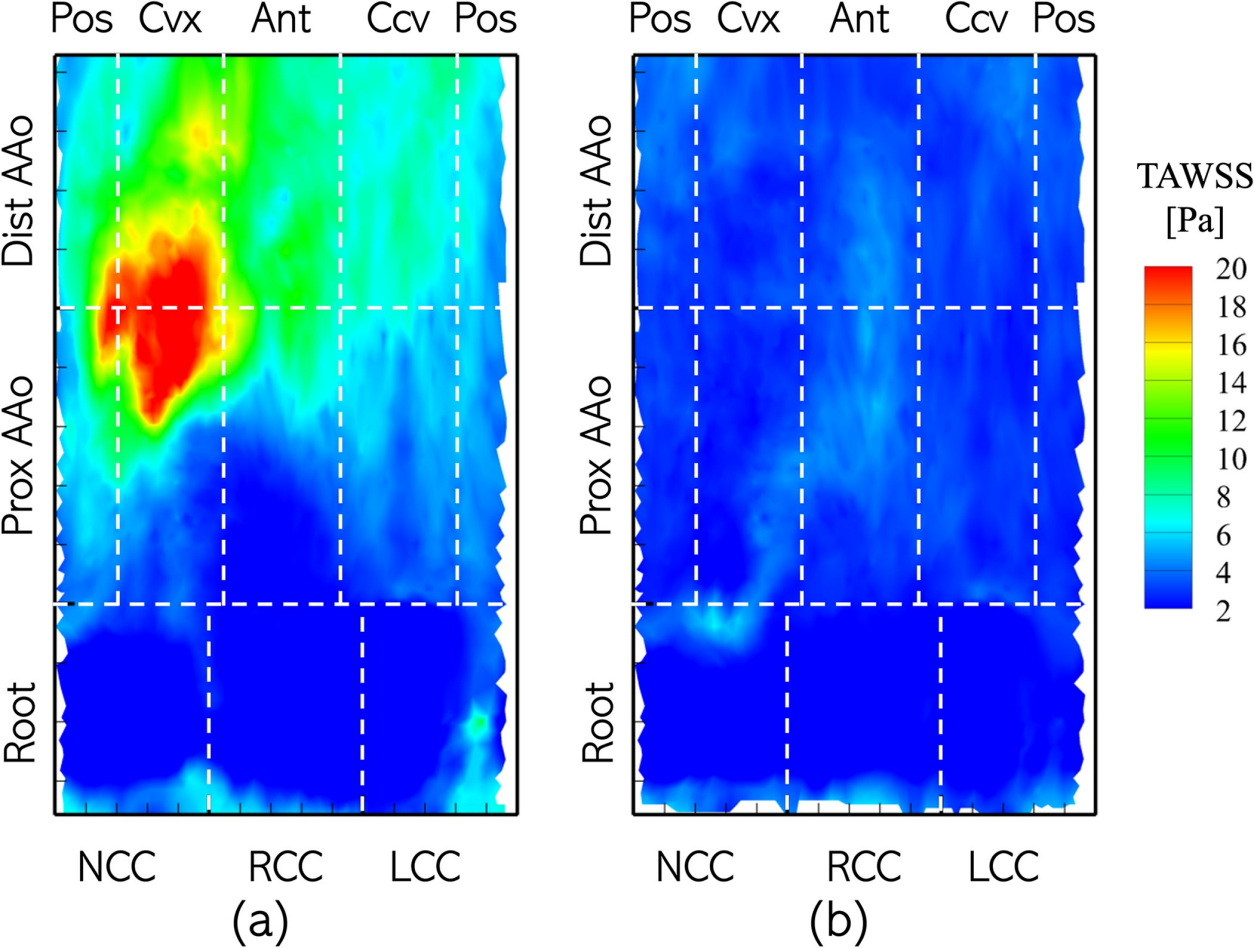
Distribution of Time-Averaged Wall Shear Stress Magnitude (TAWSS) on the aorta lumen boundary with the different analysis zones indicated using dashed lines with the Patient 1 anatomy with (a) BAV and (b) TAV. The labels along the axes indicate the location of a specific zone. Abbreviations used to identify zones of the AAo include: Pos: Posterior, Cvx: Convex, Ant: Anterior, Ccv: Concave.

In this section, the surface distribution of the four WSS-related metrics defined in section 2.5 are computed on the aorta wall in the pre- and post-intervention cases. A comparison of each metric, averaged over the patient cohort in the pre- and post-intervention scenarios, is presented for the 11 zones in which the aorta is divided as shown in Fig 4. Fig 9 illustrates the distribution of the time-averaged wall shear stress magnitude (TAWSS), calculated for the entire cardiac cycle, *T* (Eq (2)), in the (a) pre- and (b) post-intervention cases for Patient 1. Each plot may be viewed as Patient 1’s AAo longitudinally sectioned along the centerline of its posterior surface, with the horizontal axis indicating the azimuthal coordinate (θ) and the vertical axis representing the axial coordinate (*s*). Dashed lines are used to divide the surface of the aorta lumen boundary into the 11 analysis zones. The pre-intervention case is characterized by highly asymmetric distribution of TAWSS, with regions of high TAWSS (>10 Pa) localized to the convex and anterior surfaces of the PAAo and all surfaces of the DAAo. Peak TAWSS exceeded 25 Pa, observed in a streak originating on the convex PAAo and moving towards the anterior DAAo. This is attributed to the anterior shift of the high-velocity aortic jet as it travels along the lumen boundary. The root, on the other hand, is exposed to relatively low TAWSS (<5 Pa), due to weak and recirculating flow.

**Fig 10:**
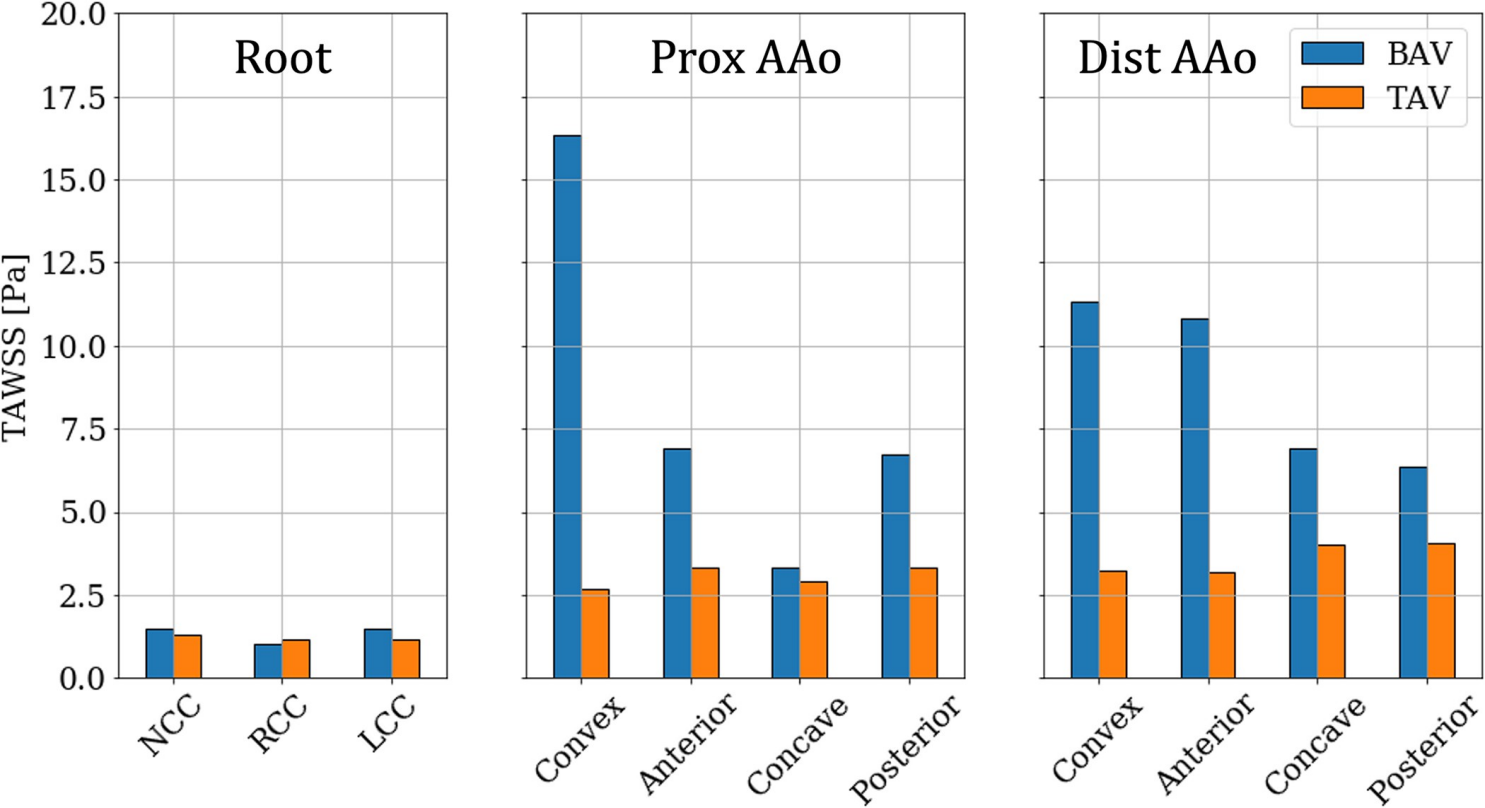
Bar plots of TAWSS averaged over individual analysis zones and the patient cohort between (blue) BAVs and (orange) TAVs.

On the other hand, the post-intervention case shows low TAWSS (<10 Pa) uniformly distributed on the lumen boundary, with most of it experiencing TAWSS ≈2 Pa. Higher stress of about 7 Pa is observed to originate at the NCC-Convex PAAo boundary, propagate into the convex PAAo, anterior PAAo and anterior DAAo, demonstrating the anterior tilting of the jet due to secondary helical flow. No appreciable differences were observed in TAWSS in the pre- and post-intervention cases, indicating weak flow velocities in the sinus cusps in both cases. Fig 10 compares the TAWSS between pre- and post-intervention cases, averaged over individual analysis zones and the patient cohort. It is observed that all cusps of the aortic root with a BAV are exposed to low TAWSS, and this state may not change with valve replacement. In the PAAo, the convex, anterior and posterior surfaces are exposed to elevated pre-intervention TAWSS (>5 Pa), with the convex surface experiencing the strongest average loading of ≈16 Pa. The concave PAAo experienced lower TAWSS of ≈3.0 Pa. On the DAAo, all surfaces experienced TAWSS >5 Pa with BAVs, with the convex and anterior surfaces experiencing similar TAWSS ≈11 Pa and the concave and posterior surfaces experiencing ≈7 Pa. The post-intervention TAWSS values on all surfaces of the PAAo and DAAo were observed to be between 2.5 & 4.0 Pa, indicating a relatively uniform distribution of hemodynamic forces on the lumen boundary with a TAV.

**Fig 11:**
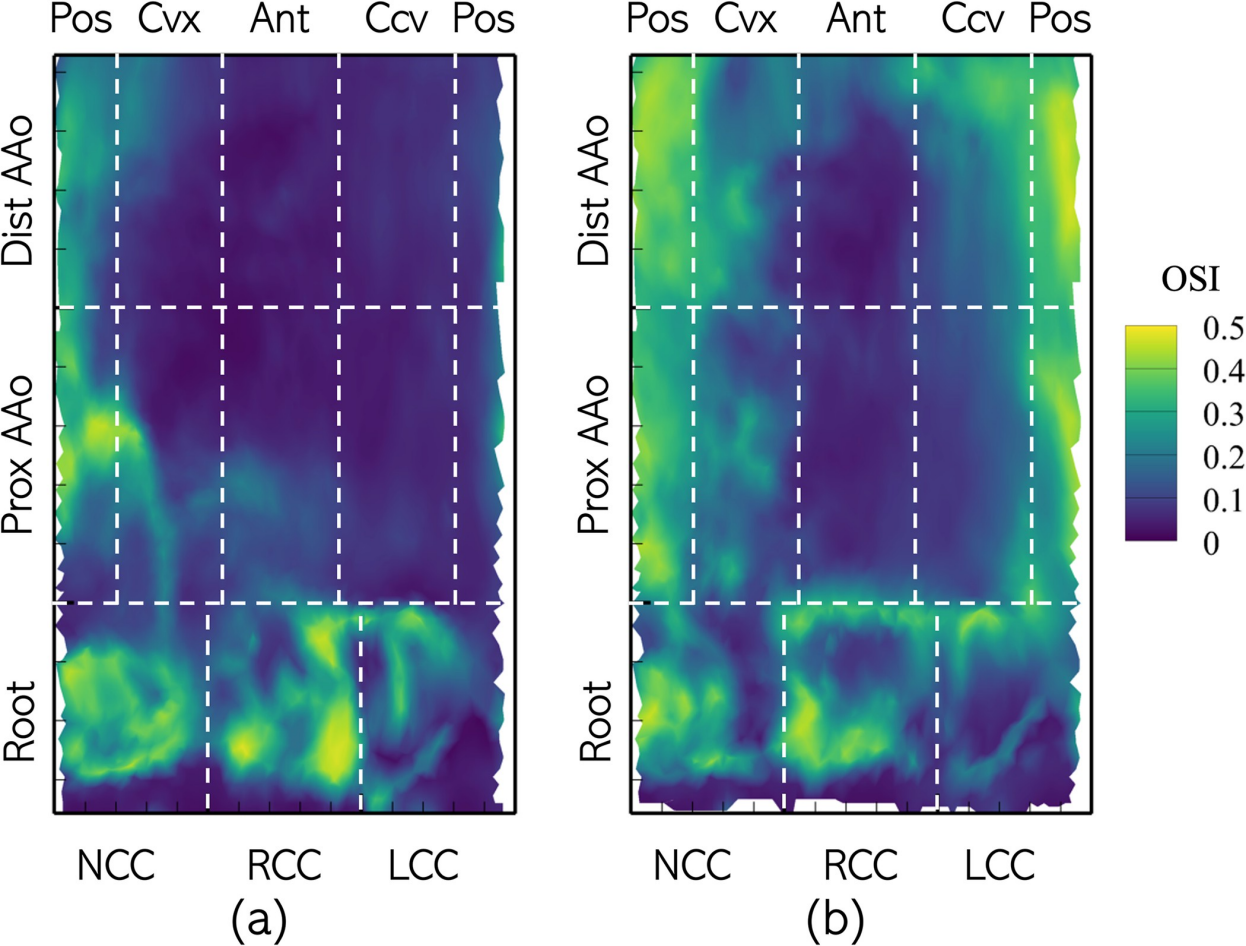
Distribution of Oscillating Shear Index (OSI) on the aorta lumen boundary with the different analysis zones indicated using dashed lines with the Patient 1 anatomy with (a) BAV and (b) TAV.

Next, the distributions of oscillating wall shear are considered using the OSI, defined as shown in Eq (3). The corresponding surface distributions of OSI on the aorta lumen boundary for Patient 1 in the pre- and post-intervention conditions are shown in Fig 11. In the pre-intervention case, all cusps of the root exhibit higher OSI in range of [0.30–0.50] with the RCC experiencing the highest of the three cusps. Most of the PAAo and DAAo experience OSI < 0.20, except for the posterior surfaces, which show OSI >0.30. Under post-intervention conditions, the root experienced similar OSI as under pre-intervention conditions, with most of the area experiencing OSI ≥0.30. Compared to the pre-intervention case, a larger area of the posterior PAAo and DAAo is exposed to high OSI. Likewise, high OSI is also observed on the convex and concave surfaces of PAAo and DAAo. Low OSI is seen on only the anterior surface of the AAo.

**Fig 12:**
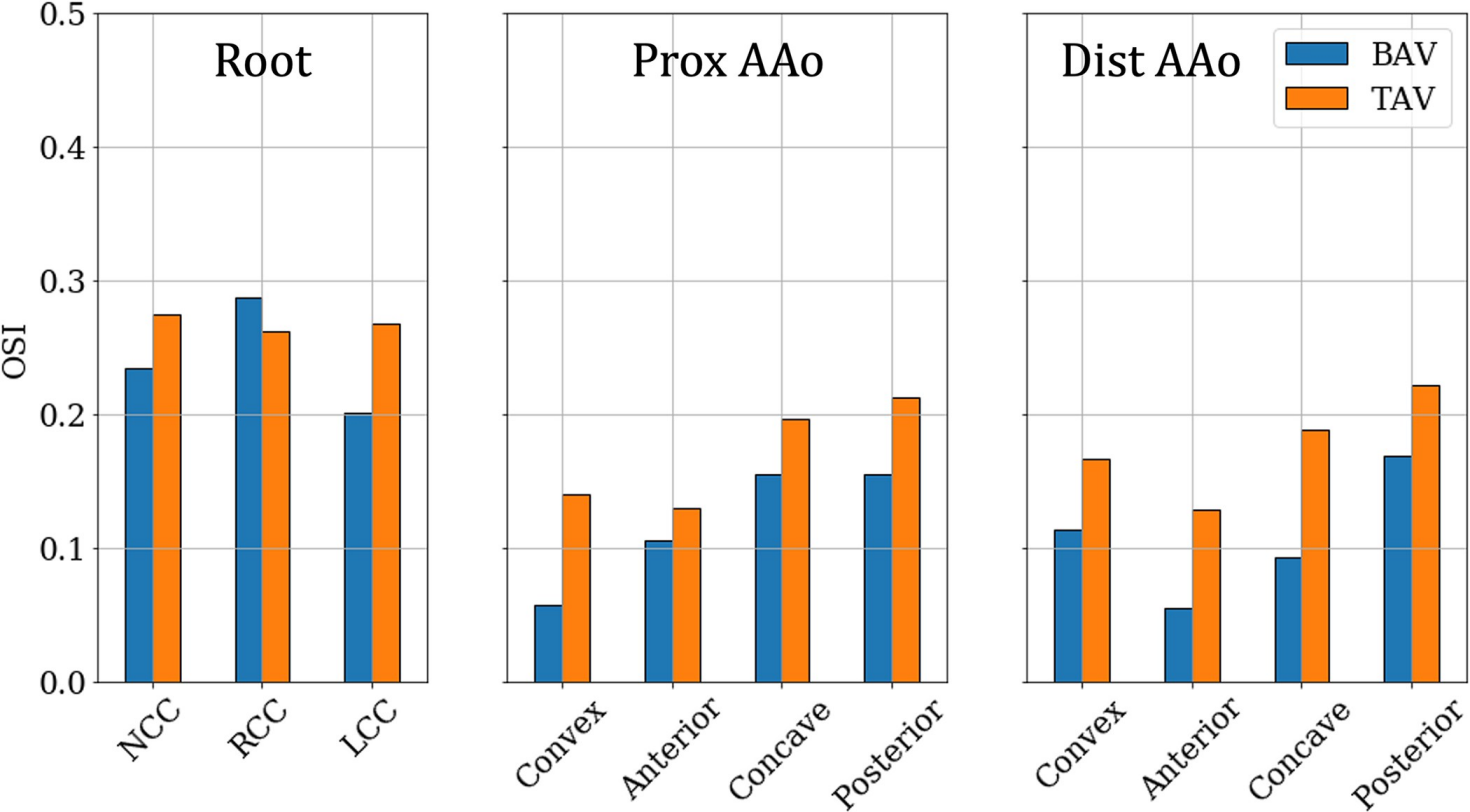
Zone-wise comparison of Oscillating Shear Index (OSI) between BAVs and TAVs.

Across the cohort, OSI in the range of [0.20‒0.30] were, on average, observed in all three cusps of the aortic root with, both, BAVs and TAVs (Fig 12). In the PAAo and DAAo, replacing a BAV with a TAV, on average, increased the OSI experienced by each zone. In the pre-intervention case, very low OSI (≤0.1) is observed on the convex and anterior surfaces of the PAAo and DAAo. The concave and posterior surfaces of the PAAo experience OSI of about 0.15 each. On the other hand, the concave surface of the DAAo experiences lower OSI (<0.1), while the posterior surface experiences marginally larger OSI ≈0.16. In the post-intervention case, each of zones in the PAAo and DAAo exhibit larger OSI compared to their respective pre-intervention values. Despite the increase in oscillating shear, the OSI in each zone was nevertheless low (<0.25).

#### 3.2.1. Divergence of wall shear stress vector

**Fig 13:**
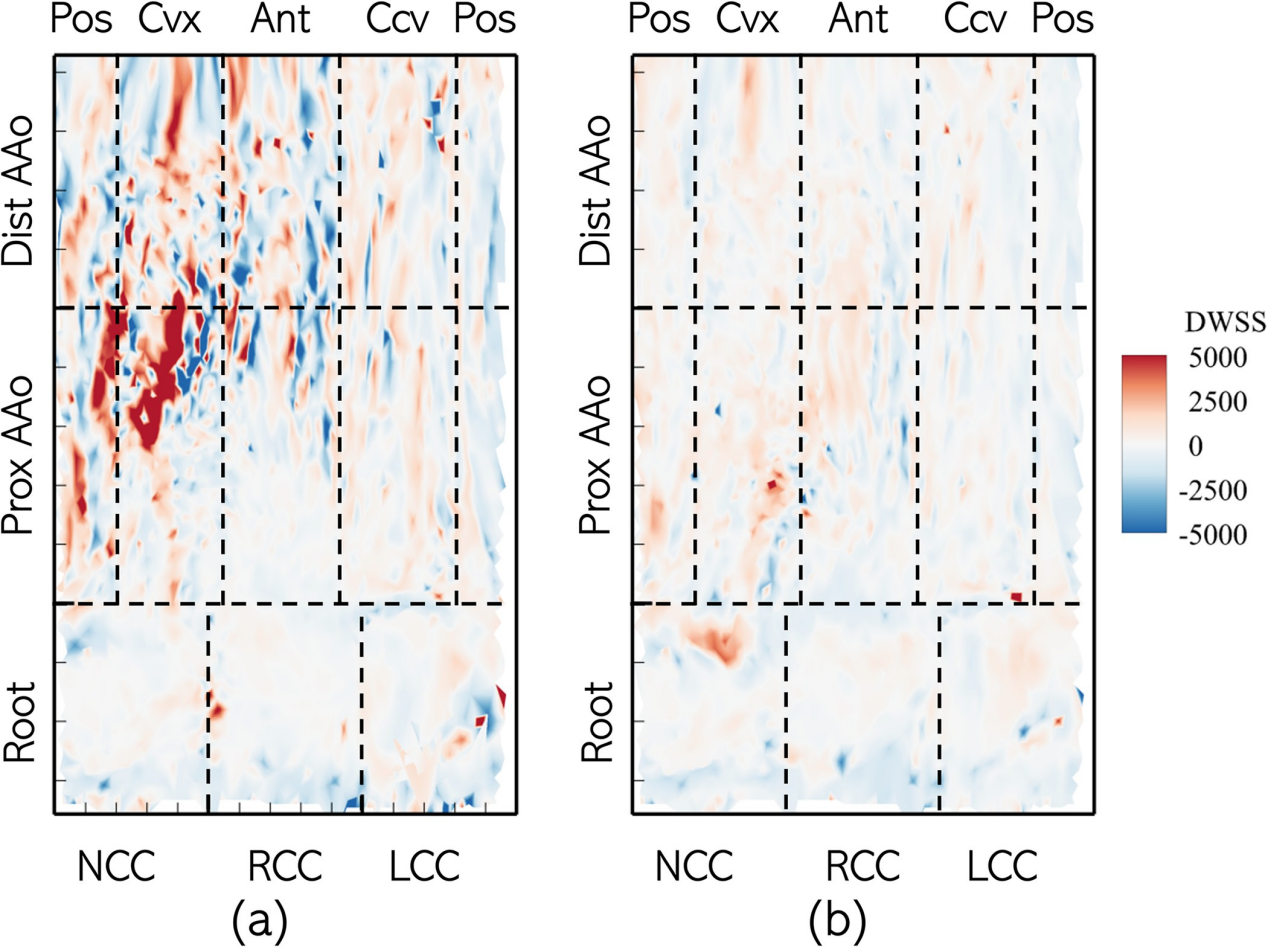
Distribution of Divergence of Wall Shear Stress Vector $\left(\nabla \cdot \vec{\tau_{W}}\right)$ on the aorta lumen boundary with the different analysis zones indicated using dashed lines with the Patient 1 anatomy with (a) BAV and (b) TAV.

The surface distributions of DWSS on the lumen boundary of Patient 1’s aorta in the two cases are shown in Fig 13. All cusps of the root experience low pre-intervention DWSS. In the PAAo, high positive DWSS (>**5000** Pa/m) streaks, moving downstream and towards the anterior surface, are observed on the posterior and convex surfaces. The remaining surfaces of the DAAo show relatively weaker DWSS streaks ($|\nabla \cdot \vec{\tau_{W}}|\leq 2500$ Pa/m). On the DAAo, the posterior, convex and anterior surfaces show narrow, alternating streaks of opposing signs. The concave surface is exposed to relatively weak DWSS. On the other hand, the post-intervention case shows low DWSS ($|\nabla \cdot \vec{\tau_{W}}|\leq 1000$ Pa/m) along the entire lumen boundary, with no remarkable streaking patterns.

**Fig 14:**
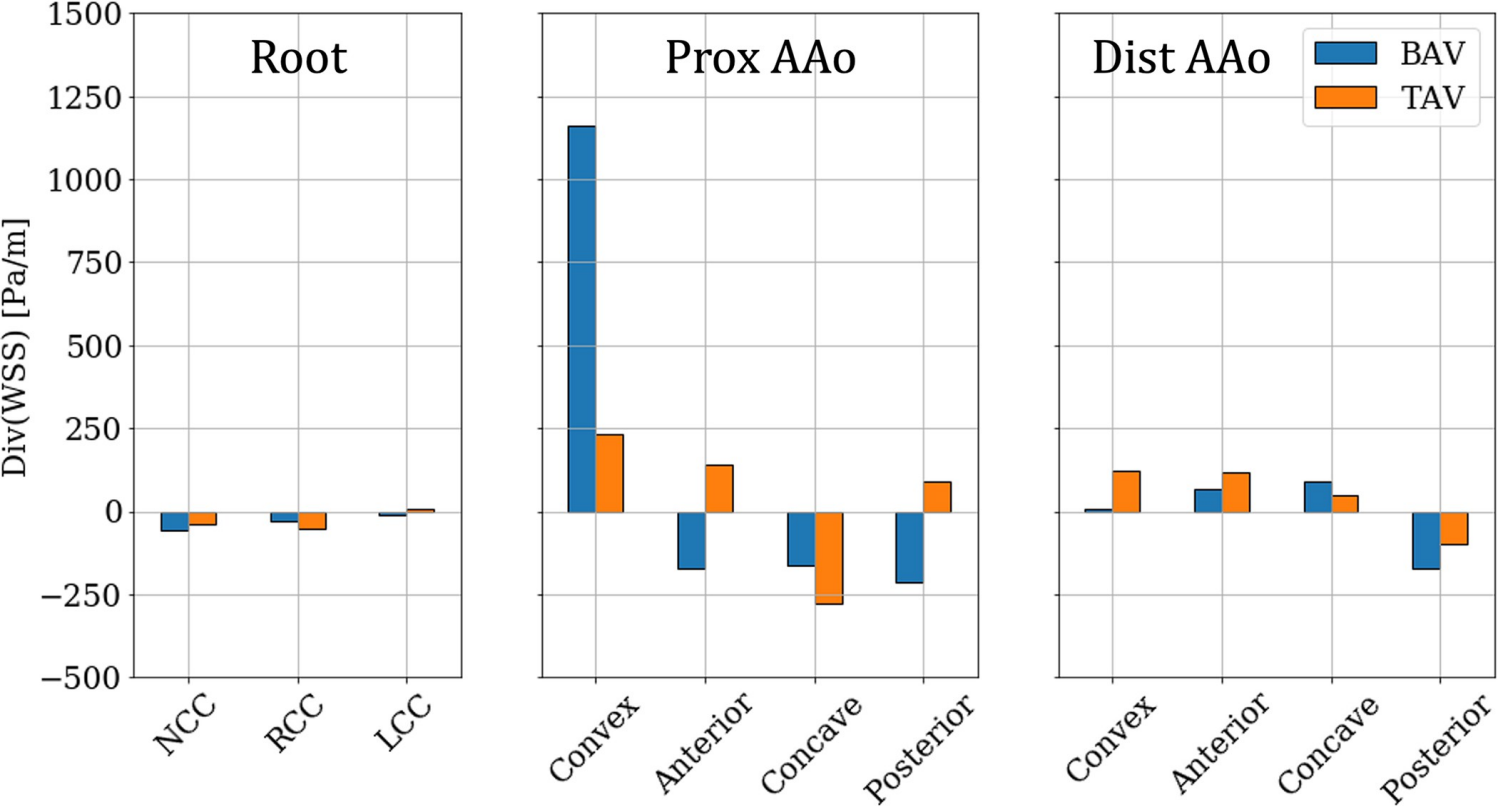
Zone-wise comparison of Divergence of WSS vector $\left(\nabla \cdot \vec{\tau_{W}}\right)$ between BAVs and TAVs.

The root experienced low DWSS (<100 Pa/m) in all cusps, across the cohort, and no significant change was observed post-intervention (Fig 14). The convex PAAo experienced the largest DWSS (>1100 Pa/m), which showed considerable resolution post-intervention where the DWSS was, on average, <250 Pa/m. The remaining three surfaces of the PAAo experienced weakly negative pre-intervention DWSS (≈‒200 Pa/m), which either changed sign, post-intervention but diminished in magnitude (anterior, posterior), or remained negative, while marginally increasing in magnitude (concave). Finally, low DWSS was observed on all surfaces of the DAAo $|\nabla \cdot \vec{\tau_{W}}|<200$, and replacement of valve with a tricuspid valve did not significantly alter this distribution.

#### 3.2.2. Endothelial cell activation potential

**Fig 15:**
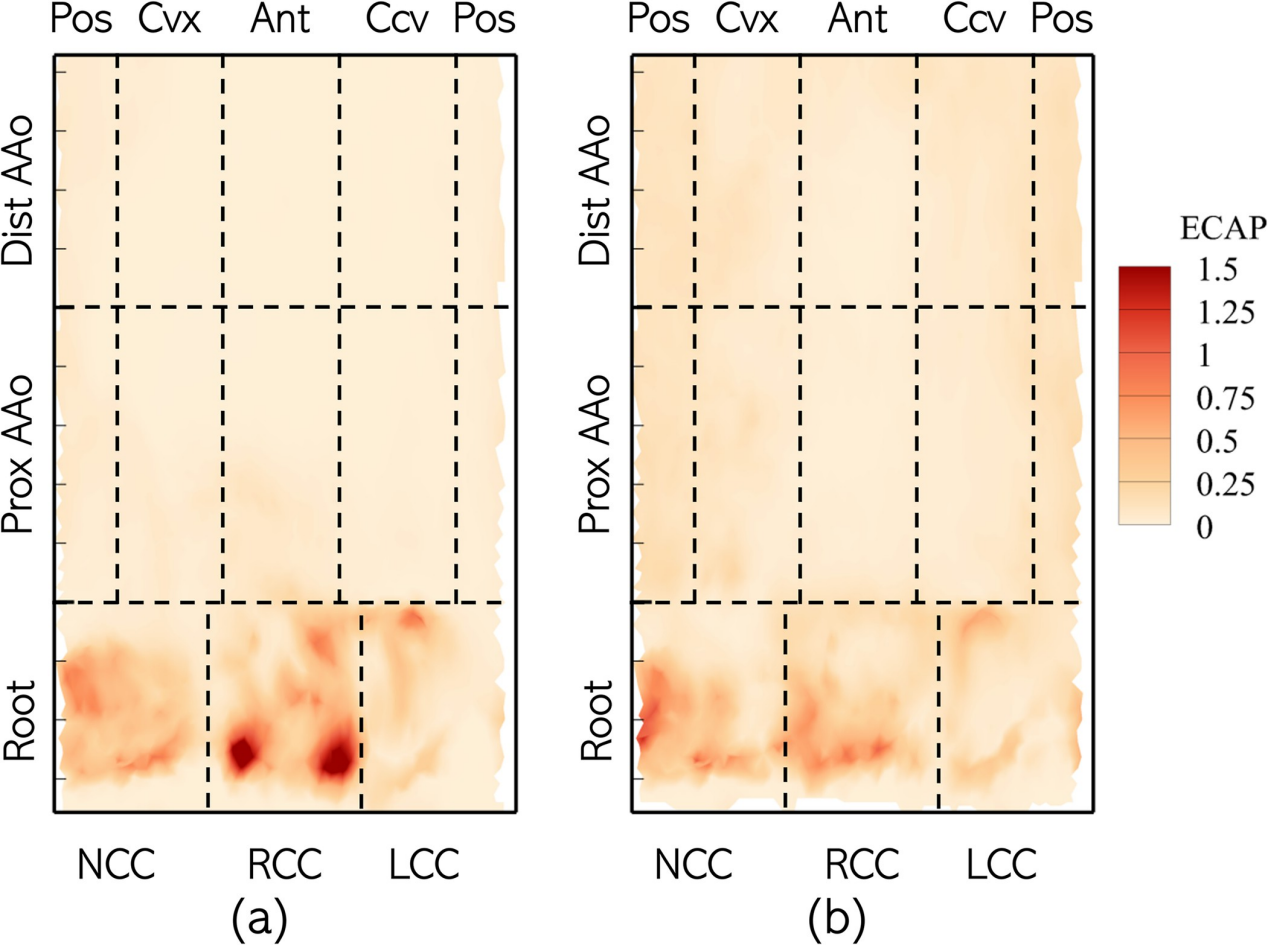
Distribution of Endothelial Cell Activation Potential (ECAP) on the aorta lumen boundary with the different analysis zones indicated using dashed lines with the Patient 1 anatomy with (a) BAV and (b) TAV.

The surface distribution of ECAP on Patient 1’s lumen boundary is shown in Fig 15. In the pre-intervention case, high ECAP (> **1.0Pa**^**‒1**^) is only observed in the root cusps, with peak ECAP (>**1.5Pa**^**‒1**^) near the base of the RCC, at the commissure lines. Both the PAAo and DAAo exhibit low ECAP (< **0.25 Pa**^**‒1**^). The ECAP distribution is not strongly affected post-intervention, with values >**1.0Pa**^**‒1**^ observed only at a few spots in the aortic root. Likewise, the lumen of the AAo only experienced low ECAP (<**0.25** Pa^‒1^).

**Fig 16:**
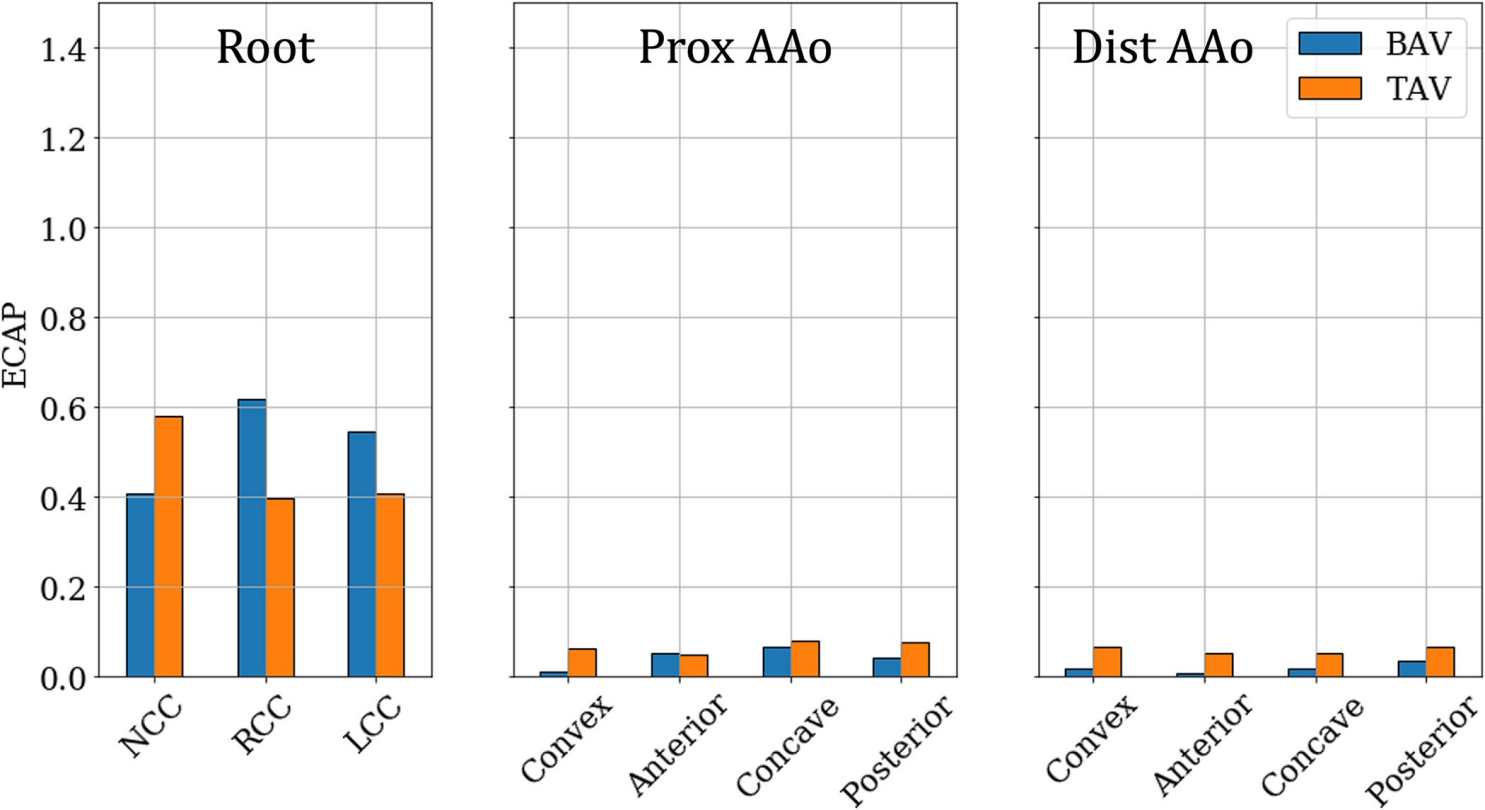
Zone-wise comparison of Endothelial Cell Activation Potential (ECAP) between BAVs and TAVs.

The observations made above for Patient 1 hold true for the whole cohort, on average: higher ECAP (≥0.4 Pa^‒1^) was only observed in the root and did not significantly change post-intervention (Fig 16). All other zones experience low ECAP (< 0.1 Pa^‒1^) in the pre-intervention case and these conditions did not change with a TAV.

## 4. Discussion

Statistical analysis of the four WSS-related metrics is performed to assess whether class wise differences in the metrics are significant using the nonparametric Mann-Whitney U test. The U-statistic measures the sum of unsigned ranks in comparisons between all possible pairs formed using one instance each from the two classes being compared in the following manner:

$$U=\sum_{i=1}^{n}\sum_{j=1}^{m}S(X_{i},Y_{j})$$ (6)

In the above equation, *S*(*X*_*i*_,*Y*_*j*_) represents pairwise comparison between the *i*^th^ and *j*^th^ elements of classes *X* and *Y* respectively, such that:

$$S(X,Y)=\left\{ \begin{array}{c} 1,\;\mathrm{if}\;X>Y\\ \frac{1}{2},\;\mathrm{if}\;X=Y\\ 0,\;\mathrm{if}\;X<Y \end{array}\right.$$ (7)

In the present case, both classes have the same number of entries, such that *m* = *n* = 6. For any WSS-derived metric, a greater disparity between the U values of the two classes, accompanied by a sufficiently small *p*-value, represents a statistically significant difference between the class ranks, indicating the individual class samples are drawn from two different populations. Distributions of the four WSS-derived metrics in each of the pre- and post-intervention classes, along with their respective *U* and *p* values in the various subzones of the Root, PAAo and DAAo are presented in Tables 2–4, respectively. Differences between pre- and post-intervention cases are considered statistically significant for *p* < 0.05, for which the null hypothesis, that the two distributions come from the same population, can be rejected.

**Table 2:** Distributions of the four WSS-derived metrics, in the pre- and post-intervention cases, and their respective U-statistic and p-values in each of the three subzones defined on the aortic root.

		TAWSS [Pa]		OSI		DWSS [Pa/m]		ECAP [Pa^-1^]	
		BAV	TAV	BAV	TAV	BAV	TAV	BAV	TAV
NCC	P1	1.27	1.45	0.28	0.23	73.46	-3.41	0.34	0.28
	P2	1.63	0.99	0.20	0.33	28.56	53.46	0.21	0.56
	P3	2.41	2.10	0.12	0.17	-126.65	-43.14	0.07	0.13
	P4	1.44	2.26	0.23	0.19	-130.56	-10.71	0.19	0.12
	P5	0.49	0.51	0.37	0.39	-109.47	-68.75	1.50	1.51
	P6	1.44	0.62	0.24	0.37	-66.50	-83.07	0.32	1.01
	*U*	19	17	16	20	13	23	15	21
	*p*	0.937		0.818		0.485		0.699	
RCC	P1	0.912	1.22	0.26	0.23	-73.19	-140.83	0.43	0.26
	P2	0.58	0.88	0.35	0.29	-17.04	-23.09	0.85	0.49
	P3	2.24	2.32	0.15	0.20	102.32	139.95	0.12	0.14
	P4	0.64	1.13	0.35	0.26	-121.48	-144.98	0.72	0.30
	P5	0.53	0.78	0.40	0.36	-49.11	-91.49	1.26	0.69
	P6	0.86	0.98	0.34	0.23	-120.44	-95.56	0.71	0.43
	*U*	8	28	25	11	22	14	28	8
	*p*	0.132		0.310		0.589		0.132	
LCC	P1	1.33	1.42	0.18	0.17	-92.06	-57.71	0.21	0.16
	P2	0.95	0.88	0.21	0.30	28.36	1.22	0.35	0.67
	P3	2.72	1.49	0.15	0.26	-238.00	208.10	0.08	0.35
	P4	1.17	1.46	0.13	0.28	113.60	47.14	0.17	0.25
	P5	0.37	0.70	0.42	0.32	-56.49	-66.43	2.33	0.63
	P6	2.46	1.04	0.13	0.28	169.58	-91.93	0.15	0.38
	*U*	20	16	8	28	17	19	10	26
	*p*	0.818		0.132		0.937		0.240	

It is observed that cusps of the root do not exhibit statistically significant differences between the pre- and post-intervention cases in terms of any of the tested WSS-related risk metrics (*p* > 0.05). However, the convex PAAo experiences statistically significant (*p* = 0.002) reductions in TAWSS, DWSS and similarly significant increases in OSI and ECAP, post-intervention. The anterior and posterior PAAo surfaces experience statistically significant reductions in TAWSS, but do not exhibit significant changes in DWSS, OSI or ECAP. While other subzones of the PAAo show some changes in the post-intervention cases, those changes are not observed to be statistically significant.

**Table 3:** Distributions of the four WSS-derived metrics, in the pre- and post-intervention cases, and their respective U-statistic and p-values in each of the four subzones defined on the proximal ascending aorta.

		TAWSS [Pa]		OSI		DWSS [Pa/m]		ECAP [Pa^-1^]	
		BAV	TAV	BAV	TAV	BAV	TAV	BAV	TAV
Cvx	P1	14.29	2.76	0.08	0.16	817.75	188.71	0.01	0.06
	P2	18.93	2.41	0.05	0.15	767.16	154.65	0.01	0.07
	P3	17.17	2.00	0.02	0.17	1662.25	260.82	0.00	0.10
	P4	20.54	2.57	0.05	0.20	1372.60	220.74	0.01	0.09
	P5	14.73	3.09	0.08	0.09	913.08	190.93	0.01	0.03
	P6	18.30	2.78	0.07	0.13	1644.23	293.14	0.02	0.05
	*U*	36	0	0	36	36	0	0	36
	*p*	0.002		0.002		0.002		0.002	
Ant	P1	4.63	3.35	0.09	0.09	-203.91	36.41	0.04	0.03
	P2	5.89	2.88	0.14	0.12	39.84	135.40	0.07	0.05
	P3	11.52	3.74	0.07	0.18	340.63	290.14	0.03	0.06
	P4	9.30	3.54	0.07	0.08	-625.19	332.32	0.04	0.03
	P5	4.34	3.06	0.15	0.16	-135.69	21.77	0.06	0.06
	P6	7.77	3.35	0.09	0.09	-354.14	95.30	0.04	0.04
	*U*	36	0	12	24	8	28	22	14
	*p*	0.002		0.394		0.132		0.589	
Ccv	P1	4.75	2.96	0.06	0.14	93.40	-19.13	0.01	0.05
	P2	3.50	3.06	0.15	0.17	-85.93	-5.97	0.06	0.06
	P3	2.13	3.93	0.23	0.21	-236.89	-1047.44	0.13	0.06
	P4	3.89	3.56	0.07	0.11	-80.29	-183.65	0.04	0.04
	P5	2.74	2.20	0.17	0.30	-115.98	-201.46	0.08	0.14
	P6	3.82	2.34	0.19	0.20	-393.77	-216.18	0.05	0.10
	*U*	22	14	12	24	20	16	14	22
	*p*	0.589		0.394		0.818		0.589	
Pos	P1	7.16	2.85	0.16	0.31	163.74	49.50	0.03	0.11
	P2	7.61	3.38	0.16	0.27	-38.79	214.22	0.04	0.08
	P3	8.93	4.55	0.13	0.05	-909.00	441.35	0.05	0.01
	P4	7.17	3.49	0.09	0.29	36.23	-39.27	0.02	0.09
	P5	4.69	2.62	0.22	0.24	-387.41	23.88	0.08	0.10
	P6	6.15	2.93	0.21	0.27	-36.65	-94.61	0.04	0.11
	*U*	36	0	6	30	11	25	6	30
	*p*	0.002		0.065		0.937		0.065	

Statistically significant decreases in TAWSS were observed on all DAAo surface subzones, while differences in OSI were significant on the anterior and concave surfaces. These differences between the PAAo and DAAo subzones correspond to the anterior tilting of the aortic jet as it moves along the aorta axis. Interestingly, all DAAo subzones show significant differences in ECAP between the pre- and post-intervention cases, although values in both classes are fairly low (ECAP < 0.1).

**Table 4:** Distributions of the four WSS-derived metrics, in the pre- and post-intervention cases, and their respective U-statistic and p-values in each of the four subzones defined on the distal ascending aorta.

		TAWSS [Pa]		OSI		DWSS [Pa/m]		ECAP [Pa^-1^]	
		BAV	TAV	BAV	TAV	BAV	TAV	BAV	TAV
Cvx	P1	11.59	3.23	0.13	0.21	22.15	64.08	0.02	0.07
	P2	11.16	3.98	0.14	0.22	37.25	242.39	0.02	0.06
	P3	14.44	3.99	0.07	0.07	-375.65	82.93	0.01	0.02
	P4	11.95	3.37	0.12	0.22	-24.86	163.06	0.02	0.08
	P5	9.12	2.20	0.15	0.13	132.08	49.39	0.03	0.08
	P6	14.04	3.05	0.08	0.17	148.07	118.77	0.01	0.06
	*U*	36	0	9	27	8	28	1	35
	*p*	0.002		0.180		0.132		0.004	
Ant	P1	10.55	3.47	0.05	0.12	-101.32	34.16	0.01	0.04
	P2	12.56	3.06	0.05	0.12	296.25	142.02	0.01	0.05
	P3	11.21	4.28	0.05	0.06	232.05	325.29	0.01	0.01
	P4	15.42	3.14	0.03	0.09	351.14	231.07	0.00	0.04
	P5	8.85	2.26	0.06	0.24	8.06	-37.25	0.01	0.12
	P6	12.12	3.28	0.04	0.10	-367.60	38.95	0.00	0.03
	*U*	36	0	0	36	17	19	0	36
	*p*	0.002		0.002		0.937		0.002	
Ccv	P1	7.62	3.80	0.06	0.20	211.19	117.09	0.01	0.06
	P2	8.21	4.21	0.06	0.11	349.25	272.14	0.01	0.03
	P3	6.58	4.86	0.11	0.13	-54.85	-170.67	0.02	0.03
	P4	9.32	4.43	0.05	0.10	-26.33	2.11	0.01	0.03
	P5	5.46	2.93	0.14	0.33	-3.76	16.53	0.03	0.11
	P6	5.51	3.90	0.10	0.21	-39.54	65.57	0.02	0.05
	*U*	36	0	5	31	17	19	1	35
	*p*	0.002		0.041		0.937		0.004	
Pos	P1	8.29	3.87	0.16	0.33	-205.87	-84.20	0.02	0.09
	P2	6.48	4.77	0.13	0.20	272.88	9.76	0.02	0.05
	P3	6.46	5.64	0.19	0.08	-408.26	-191.42	0.04	0.02
	P4	7.52	4.41	0.10	0.22	-217.13	-200.47	0.01	0.05
	P5	5.20	2.60	0.20	0.26	-249.07	0.62	0.05	0.11
	P6	5.40	3.71	0.17	0.28	-202.90	-153.49	0.04	0.08
	*U*	34	2	6	30	11	25	5	31
	*p*	0.008		0.065		0.937		0.041	

The data indicate that BAVs generate strong, eccentric, jets which tilt in the direction of, and attach to the convex surface of the proximal AAo. This occurs somewhat earlier than jets observed with TAVs. The severely stenotic BAVs result in peak gradients exceeding 100 mmHg and consequent peak jet velocities over 4 m/sec. The eccentric, high-speed jet and early attachment result in significant positive (or right-handed) flow helicity and anterior tilting of the jet in the AAo. Because of this preferential attachment of the jet to the convex and anterior aortic surfaces, the pre-intervention cases show localized supraphysiological TAWSS (> 20 Pa) originating at the convex PAAo and radiating downstream and towards the anterior surface. The pre-intervention gradients and jet velocities agree with corresponding expected values for the degree of induced stenosis. Barker et al. [58] reported that type 1 BAVs with RL-fusion are associated with significantly elevated WSS at focal regions in the AAo, and these regions correspond to the aortic jet impingement locations. Sotelo et al. [27] have reported, through 4D flow MRI-based measurements, that BAV patients exhibit strong, positive axial circulation in the AAo which extends until the aortic arch. In comparison, healthy volunteers exhibited considerably weaker positive axial circulation. These observations are corroborated by our results: the pre-intervention helicity flux integral is strongly positive at all locations except near the sino-tubular junction (section 1). It reaches a peak value in mid-AAo and decreases distally towards the arch. In contrast, helicity flux was relatively uniform across all AAo sections in the post-intervention simulations.

In another study by Sotelo et al. [28], BAV patients were found to have a 220% increase in circumferential WSS (WSSc), compared to healthy volunteers. This increase in WSSc is a direct consequence of the strong helical flow in the AAo and was found by Minderhoud et al. [31] to be a strong predictor of AAo dilation. While WSSc is not analyzed in this study, some information about circumferential forces may be inferred from DWSS. The convex proximal AAo experiences large positive DWSS, indicating that the local WSS vectors point outwards so that WSS acts to locally stretch the lumen, increasing the risk of vascular injury. The aortic root, on the other hand, experienced TAWSS ≈ 2 Pa in each cusp, with low DWSS. The post-intervention TAWSS distribution shows significant resolution of pathological wall loading and is characterized by TAWSS < 5 Pa uniformly distributed on the PAAo and DAAo. No significant changes to TAWSS or DWSS are observed in the aortic root. Thus, valve replacement may significantly improve hemodynamic loading on the AAo wall, but not on the cusps of the aortic root.

The stronger and highly directional aortic jets secondary to BAVs result in low OSI (≤ 0.1) on the convex and anterior surfaces of the PAAo and the anterior and concave surfaces of the DAAo. On the other hand, the weaker, more diffused jets associated with healthy TAVs, result in OSI in the [0.1‒0.2] range on all surfaces of the PAAo and DAAo. In the root, the pre- and post-intervention OSI were similar and in the [0.2‒0.3] range.

**Fig 17:**
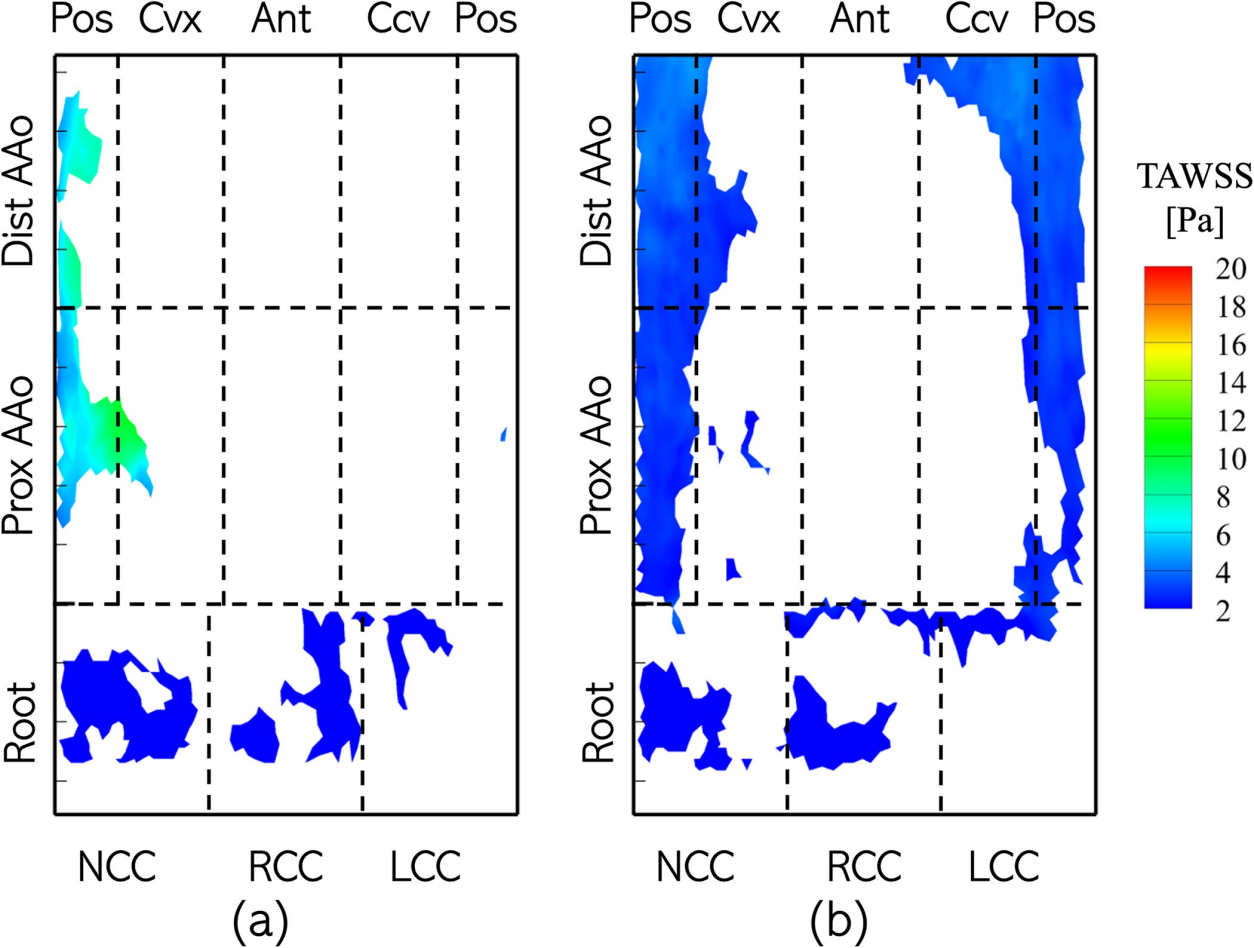
Distribution of TAWSS in regions on the aorta lumen boundary experiencing OSI >0.25 for Patient 1 in the (a) pre-intervention and (b) post-intervention cases.

The ECAP distributions suggest that patients presenting with stenotic BAVs might be at lower risk for AAo aneurysm, but higher risk for aortic root aneurysm and that these risks might not be mitigated post AVR. By definition, ECAP favors regions experiencing high OSI and low TAWSS, i.e. low, and oscillating wall shear. To further investigate the possible role of low and oscillatory WSS in BAV-related aortopathy, the zones experiencing high OSI (>0.25) were isolated in the pre- and post-intervention cases for Patient 1, and the TAWSS experienced by those regions analyzed. We observed that in the pre-intervention case (Fig 17A) low-OSI regions primarily exist in the root cusps and posterior AAo. Of these, only the aortic root was subject to low TAWSS. On the contrary, larger areas of the root and AAo were exposed to both high OSI and low TAWSS in the post-intervention case (Fig 17B).

**Fig 18:**
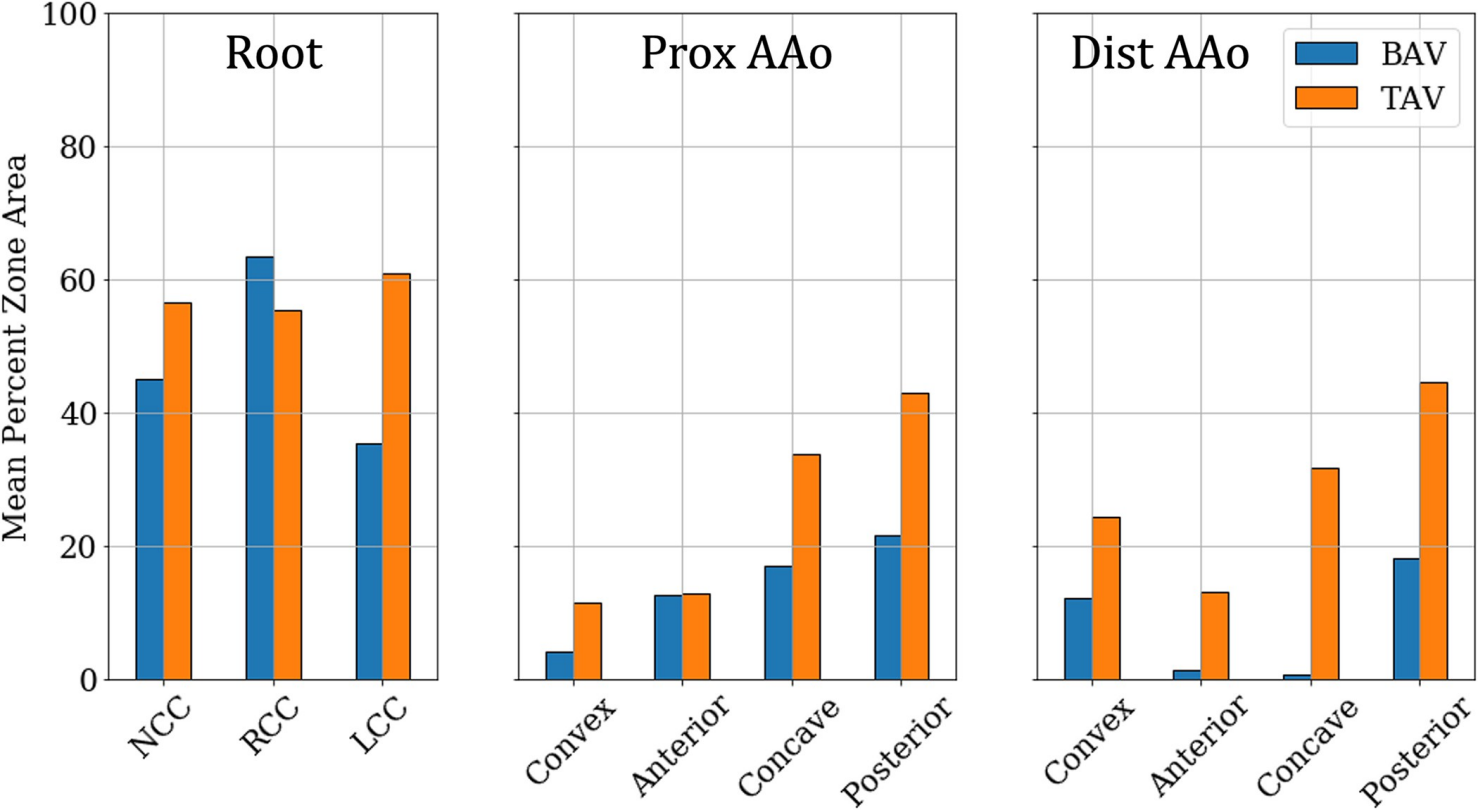
Zone-wise comparison of surface areas exposed to OSI >0.25, averaged over the patient cohort, in the pre- and post-intervention cases.

The trend of a larger part of the AAo exposed to higher oscillating WSS post-intervention was observed across the patient cohort, as seen in Fig 18. Each zone in the post-intervention AAos has a larger surface area, on average, experiencing OSI >0.25, compared to those with BAVs, with significant increases observed particularly in the DAAo. Moreover, valve replacement is associated with lower TAWSS (Figs 9 and 10) and tensile surface forces, measured using DWSS (Figs 13 and 14), over the entire AAo lumen boundary. If low and oscillatory WSS is indeed the predictor for aortopathy, these observations appear to imply that individuals with healthy TAVs may not be at a lower risk for AAo dilation and dissection, compared to those with severely stenotic BAVs. It may also be inferred that valve replacement might provide no significant benefit to the risk of root aneurysm formation. However, these conclusions are not supported by clinical observations [12–14,59], and therefore, cast doubt on the correlation between low and oscillating WSS and aortopathy. Thus, investigating an alternative hypothesis is warranted.

**Fig 19:**
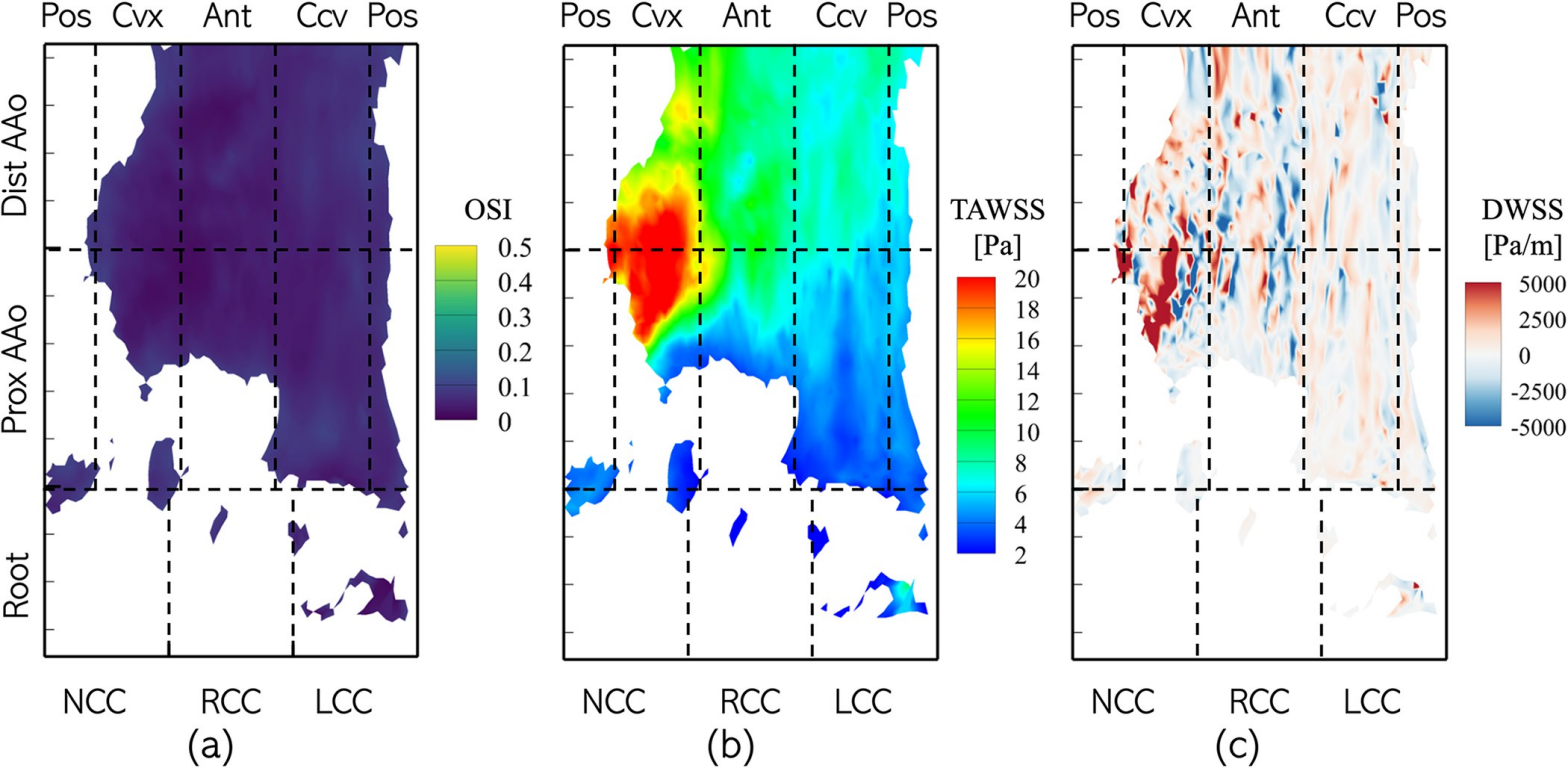
Distribution of (a) OSI (b) TAWSS and (c) DWSS on parts of Patient 1’s pre-intervention aorta lumen boundary experiencing low OSI (<0.1).

The results presented herein confirm the findings of Dux-Santoy et al.[26] suggesting that hemodynamics resulting due to BAV are not associated with low and oscillating TAWSS in the AAo (Figs 10 and 14); on the contrary, strong and unidirectional, eccentric aortic jets lead to high TAWSS/ low OSI on large parts of the AAo wall. Fig 17 shows that high OSI regions generally matched with those experiencing low TAWSS, but these regions, on average, made up less than 20% of the AAo surface area with BAVs (Fig 18). On the other hand, regions exhibiting very low OSI (≤0.1) coincided with those experiencing large TAWSS and positive DWSS streaks (Fig 19). Regions with such abnormally low OSI and strong WSS were found particularly on the convex surface of the PAAo and the convex and anterior surfaces of the DAAo. A similar observation was made by Cao et al. [39], wherein, the authors simulated blood flow in an idealized AAo, driven by physiological ventricular function, with a healthy tricuspid valve and three different type-1 BAV phenotypes. With LR-BAVs, the authors report similar jet tilting and skewness towards the convex wall of the AAo as seen in section 3.1. The authors found that the convex and concave surfaces of the AAo experienced similar levels of TAWSS but were differentiated in terms of their exposure to low OSI; the convexity was exposed to abnormally low OSI (≈0.1), whereas the concavity experienced OSI in the [0.2‒0.4] range. The regions with combined low OSI and high TAWSS coincide with those typically at high risk of dilation in patients with stenotic BAVs [13,14,29].

The results and statistical analysis presented herein show significant differences in the WSS-derived hemodynamic metrics on focused regions of the PAAo and DAAo, which coincide with those clinically observed to be susceptible to pathological growth rates. Strong aortic jets which affect preferentially some regions of the lumen boundary and their tilting due to the natural AAo curvature and concomitant helical flow are implicated in the formation of pathological hemodynamic conditions (bicuspid aortopathy), which may contribute to aortic enlargement. Although there is not yet convincing evidence that valve replacement may impact the evolution of the concomitant aortopathy, the post-intervention simulations suggest that such conditions may be alleviated via valve replacement. Thus, if the pre-intervention WSS trends are representative of the hemodynamic aspect of ascending aortic dilatation, valve replacement may prevent further acceleration of the aortic diameter growth. Larger studies with long-term follow-up may help establishing this cause-effect relationship. The main intent of our study is to gather preliminary evidence of such relationship.

Interestingly, despite vastly different pre- and post-intervention hemodynamics in the ascending aorta, replacing a dysfunctional valve did not appear to induce significant differences in any WSS-derived metric within the aortic root. It may be inferred that the WSS patterns in aortic roots with severely stenotic BAVs either do not contribute to the hemodynamic aspect of root dilation or, if they do, that root dilation rate may not be significantly arrested after valve replacement. Clinical observations have found the presentation of root dilatation to be unrelated to the presence & severity of BAV stenosis. Rather, its occurrence is associated with the presence of regurgitant BAVs.

Considering these findings, it may be important to carefully monitor AAo hemodynamics during TAVR planning, and to estimate dilation risk for severely stenotic BAVs. Likewise, a priori estimation of the effect of valve replacement on aortic jet dynamics and the consequent WSS on the aorta can provide valuable preprocedural planning support. Unfortunately, transthoracic or transesophageal echocardiography routinely employed in TAVR planning lacks spatio-temporal resolution to accurately characterize the desired flow features. Pre-procedural flow may be better quantified using 4D flow MRI, but such imaging is frequently inconvenient to the patient and much more expensive, furthermore it may prove beneficial only for a small fraction of TAVR cases and cannot provide a priori estimation of post-procedural hemodynamics. Recently, simulation-based transcatheter heart valve replacement planning has emerged as a cost-effective supplement to imaging-based planning (FEops HEARTguide, Ghent, Belgium; DASI Simulations, OH, USA), but it is largely limited to structural heart modeling and is yet to be widely adopted. Incorporating patient-specific fluid-structure interaction modeling into such analyses can augment their capability to predict hemodynamics-related complications. Further development of the present methodology can provide an efficient way to simulate pre- and post-procedural hemodynamics and enable a wide range of hemodynamics-related risk prediction.

## 5. Conclusion

This study uses computational modeling to study the effect of severely stenotic bicuspid aortic valves on AAo hemodynamics, with the aim of quantifying hemodynamic metrics that are known to correlate with the risk of AAo dilation or dissection. We also use these computational models to make a virtual comparison between pre- and post-valve replacement aortic hemodynamics. The role of WSS magnitude and directionality in the potential risk of aortic dilation and dissection are assessed by quantifying the distribution of four WSS-derived metrics on key zones constituting the aorta anatomy and observing changes in each metric post-intervention.

The major findings of this study may be summarized as follows:

1. Aortic jets associated with stenotic BAVs show high eccentricity and peak flow velocity (> 4 m/s). The eccentric jets result in secondary helical flow and regionally asymmetric jet on the AAo lumen boundary. The flow constriction due to a stenotic valve also results in large peak gradients across the valve.
2. Patients with severely stenotic BAVs receiving therapeutic intervention via bioprosthetic valve replacement may experience improved hemodynamics in terms of reduced aortic jet velocity, transvalvular gradients, jet eccentricity, flow helicity and turbulence.
3. BAVs generate regionally asymmetric distributions of WSS with the proximal and convex surfaces of the AAo experiencing abnormally large (>10 Pa) WSS. The corresponding WSS distribution with TAVs is more uniform with low magnitude (<5 Pa).
4. For BAVs, regions experiencing high TAWSS coincide with those experiencing abnormally low OSI (< 0.1). Conversely, regions experiencing high OSI (> 0.25) coincide with those exposed to lower TAWSS. However, these regions comprised of, on average, < 20% of the AAo lumen boundary. On the other hand, post-intervention, a larger fraction of the AAo lumen boundary, and nearly half of its posterior surface, experienced oscillating WSS.
5. A large portion of the pre-intervention AAo lumen boundary is exposed to abnormally low OSI (<0.1) and it includes regions experiencing strong and diverging WSS. Such regions are absent in the post-intervention cases.
6. For BAVs, the convex PAAo is also exposed to large positive DWSS, indicating strong, localized tensile forces. DWSS is either low or negative on all surrounding regions (anterior/ posterior PAAo/ convex DAAo) indicating a large local change in the nature of the biomechanical loading on the lumen boundary from tensile to compressive and a greater risk for tissue damage. Such loading conditions were not observed in the post-intervention cases, indicating a possible slowing down of disease progression.
7. ECAP was observed to be low across the surface in both the pre- and post-intervention cases and showed significant differences in regional distributions, particularly in the DAAo. Previous studies [17] determined a threshold of 1.4 Pa^-1^ for increased risk of endothelial damage, but this threshold is not met in any simulated case. Therefore, there is insufficient information from the current study to comment on the suitability of ECAP as a predictor of aortic dilation.

This study is characterized by several limitations which stem from challenges in sampling, modeling, and parametric testing. For instance, recruiting more patients in the cohort can improve the statistical power of the analysis of average metrics. Expanding the patient cohort to include up to O(100) patients would require considerable development of our solvers and acceleration of individual simulations. The effect of WSS on the endothelial response, the consequent risk for vascular remodeling, and finally, aortic dilation or dissection are not directly modeled. Doing so would require simulating the underlying microscale phenomena over long periods of time, extending into months or years. The near wall flow and, consequently, wall loading are affected by compliance of the aorta walls, leading to systolic dilation in every cardiac cycle. These effects are not modeled and may affect the accuracy of the results. The rDOF valve model employed herein may not accurately resolve all transient dynamics of leaflet motion, but these effects may be small, and within the overall uncertainly of the modeling procedure. The same synthesized flow-rate profile was used for all patients so we are not able to precisely reproduce the hemodynamics occurring in vivo. Another limitation of the current model is the Newtonian approximation for blood rheology. It is known that blood is a suspension of red and white blood cells in a largely Newtonian plasma, and consequently, blood exhibits shear-thinning properties. Such effects were first observed in vessels of diameters < 0.3 mm [60], which was historically used to justify employing Newtonian rheology in larger vessels. Recent computational studies have employed different mathematical models to test the effect of non-Newtonian rheology on hemodynamics in major arteries. However, despite the increased interest, the extent to which non-Newtonian effects influence WSS remains unclear, with some reporting a significant impact [61–63] and others concluding that the effect is relatively weak [64,65]. Thus, accounting for the shear-thinning effect of blood on mechanical stresses may be important in the current application and can be done as part of a future investigation. The study uses one pre-intervention case for clinical validation of simulations but does not present any post-intervention validation with the prosthetic valve, due to unavailability of post-procedural echocardiographic data. Finally, the post-implant case does not actually model the deployment of these valves and the changes in the aortic root that occur due to deployment. The BTAV cases also do not include other features of the implants such as stents, skirts, annular rings. Despite these limitations, the study provides new insights into the pathological hemodynamics associated with severely stenotic BAVs and provides quantification of changes in hemodynamics that results from valve replacement. Given the fidelity of the fluid model employed here, coupling of the current hemodynamics model with a transcatheter valve deployment simulator [66] could lead to a powerful tool for better understanding the effect of aortic valve replacement procedures on aortic hemodynamic changes.

## Supporting information

S1 Appendix Fluid-structure interaction modeling of the valve. (DOCX)

S2 Appendix Adaptation of valve model to patient-specific anatomy. (DOCX)

S3 Appendix Solution verification. (DOCX)

S4 Appendix Model validation. (DOCX)
