# Supplementary material for: Changes in aorta hemodynamics in Left-Right Type 1 bicuspid aortic valve patients after replacement with bioprosthetic valves: An in-silico study
Source: PLoS One. 2024 Apr 16;19(4):e0301350. doi: 10.1371/journal.pone.0301350 (PMC11020955; doi:10.1371/journal.pone.0301350)
Supplement: S1 Appendix — (DOCX) [file pone.0301350.s001.docx]

# Supplementary Data

# SA Appendix: Fluid-Structure Interaction Modeling of the Valve

Since the focus here is on transvalvular and ascending aorta hemodynamics and not the detailed mechanics of the valve leaflets, some simplifications in the aortic valve model can be employed to enhance the computational efficiency of these simulations. Here, we employ a versatile reduced degree-of-freedom (rDOF) leaflet model wherein leaflet motion is governed by the following equation of motion:

|  | $\alpha\frac{\partial\vec{v}_{v}}{\partial t}=\Delta p\vec{n}-\kappa\left( \vec{d}_{v}-\vec{d}_{v,0} \right)$ | (S |
| --- | --- | --- |

where, $\vec{d}_{v}$ and $\vec{v}_{v}$ represent instantaneous leaflet position and velocity, respectively, and $\vec{d}_{v,0}$ is the fully closed leaflet configuration. The valve model is parametrized using two constants, $(\alpha,\kappa)$ relating to leaflet mass and stiffness,­_­­_­_­_ such that equation (S1) describes a balance between leaflet inertia ($\alpha$), driving pressure difference across leaflet surface ($\Delta p$), and restoring forces due to linear tissue elasticity ($\kappa$). Instantaneous leaflet displacement is expressed in terms of a range-of-motion vector, $\vec{b}\left( \vec{x} \right)$, and a mapping function $\xi\left( \vec{x},c\left( t \right) \right)$, as shown in equation (S2).

|  | $\vec{d}_{v}\left( \vec{x},t \right)=\vec{b}\left( \vec{x} \right)\xi\left( \vec{x},t \right)$ | (S |
| --- | --- | --- |

The range-of-motion vector is defined for each point on the leaflet surface as the difference between its coordinates at maximally open and closed configurations (equation (S3)). An illustration of the same is shown in S1 Fig for the free end of one leaflet centerline (shown using the dark black line in S1 Fig (a)). The motion of the centerline over several phases during valve opening is shown in S1 Fig (b). From these, the fully open and closed leaflet configurations are isolated in S1 Fig (c), indicating the vector $\vec{b}\left( \vec{x} \right)$ for its free edge.

|  | $\vec{b}\left( \vec{x} \right)=\vec{x}_{open}-\vec{x}_{close}$ | (S |
| --- | --- | --- |


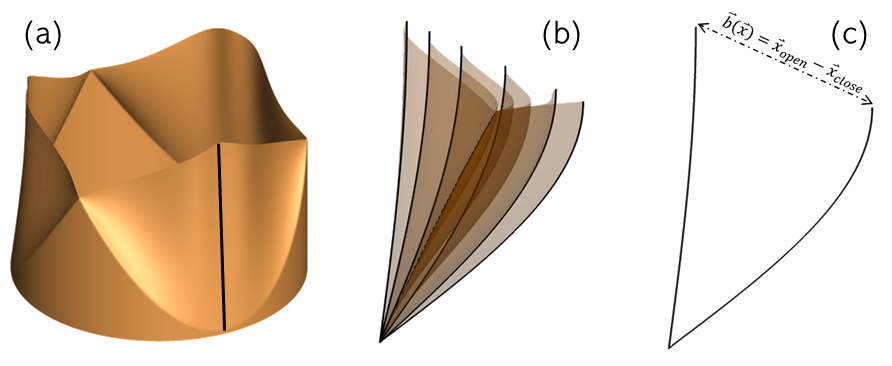


S1 Fig: (a) Idealized valve model with circular cross-section with leaflet centerline highlighted using a black line, (b) snapshots of leaflet centerline at several instances during valve opening and (c) leaflet centerline at maximally opened and closed configurations, indicating the local range-of-motion vector, $\vec{b}\left( \vec{x} \right)$.

The mapping function $\xi$ is scalar-valued and depends on the location on the leaflet surface and the instantaneous phase in the cardiac cycle, via a lumped displacement $c\left( t \right)$ [$0<c\left( t \right)<1$]. The purpose of the mapping function is to compute the instantaneous leaflet displacement via interpolation on the range of motion vector using the lumped displacement and appropriate distribution of the same over the leaflet surface to obtain desired leaflet kinematic features. We assume leaflet motion is comprised of large- ($\xi_{LS}\left( \vec{x},t \right)$) and fine-scale ($\xi_{f}\left( \vec{x},t \right)$) features as shown in equation (S4):

|  | $\xi\left( \vec{x},t \right)=\xi_{LS}\left( \vec{x},t \right)+\xi_{f}\left( \vec{x},t \right)$ | (S |
| --- | --- | --- |

Here, $\xi_{LS}$ represents the opening/ closing motion of individual leaflets and $\xi_{f}$ represents systolic flutter. The large-scale features govern leaflet opening/ closing mode shapes. To replicate commonly observed valve shapes, we tested two mapping functions, one depending linearly, and the other through a power-law relation, on $c\left( t \right)$, as seen in equations (5) (a) and (b), respectively. The corresponding leaflet mode shapes are illustrated in S2 Fig (a) and (b).

|  | $\xi_{LS}\left( \vec{x},t \right)=c\left( t \right)$ | | (a) |
| --- | --- | --- | --- |
|  | $\xi_{LS}\left( \vec{x},t \right)=c\left( t \right)^{\beta\left( \vec{x} \right)}$ | (S (b) | |
|  | $\xi_{f}\left( \vec{x},t \right)=\Xi\left( \vec{x} \right)\frac{c\left( t \right)}{c_{max}}f_{\theta}\left( \vec{x} \right)f_{ax}\left( \vec{x},t \right)$ | | (c) |


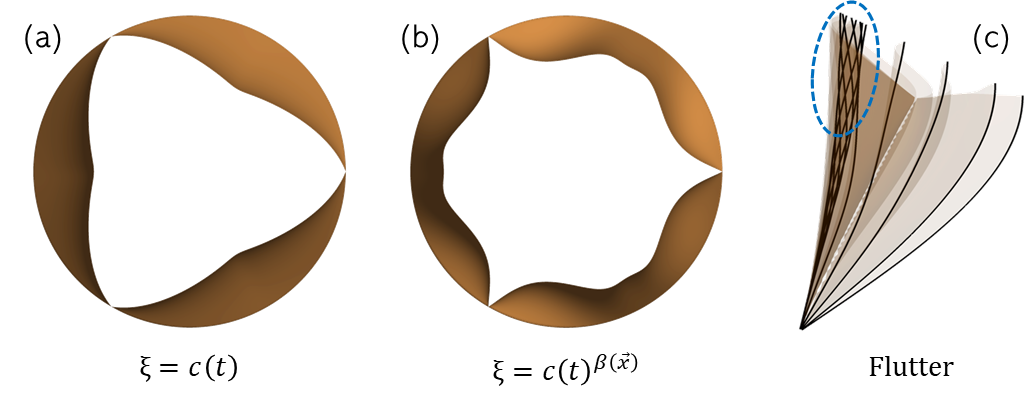


S2 Fig: Examples of leaflet kinematic features which can be obtained by tuning the mapping function,$\xi$: commonly observed opening mode shapes can be obtained using simple mathemtical dependencies such as (a) linear and (b) power relations between the mapping function and lumped displacement. Higher-order kinematic features like leaflet flutter can also be incorporated in leaflet motion (c) by including a fine-scale component in$\xi$.

Leaflet flutter is modeled by superimposing high-frequency harmonic functions on the large scale motion. These harmonic functions have azimuthal and axial components $f_{\theta}\left( \vec{x} \right)$ and $f_{ax}\left( \vec{x},t \right)$ respectively in equation (S5) (c), where the former is modeled as a standing wave and the latter as a traveling wave. We neglect the azimuthal component flutter by setting $f_{\theta}\left( \vec{x} \right)=1$. Further, flutter amplitude is defined as a fraction of the instantaneous lumped displacement using $\Xi\left( \vec{x} \right)$, which can be reasinably set to 5-10%, such that leaflet flutter is maximal at the fully open configuration and vanishes in its closed position. The resulting leaflet flutter is illustrated in S2 Fig (c).

Differentiating the ansatz for leaflet position we get an expression for the local velocity, as shown in equation (S6).

|  | $\vec{v}_{v}\left( \vec{x},t \right)=\frac{dc}{dt}\left( t \right)\frac{\partial\xi}{\partial c}\left( \vec{x},c\left( t \right) \right)\cdot\vec{b}\left( \vec{x} \right)$ | (S |
| --- | --- | --- |

Next, we substitute the displacement and velocity ansatz in the normal component of the leaflet equation of motion (equation (S1)) and integrate over leaflet surface to obtain the following second-order ODE for the lumped displacement:

|  | $\frac{d^{2}c}{dt^{2}}=\frac{F_{P}-F_{S}-F_{m,V}-F_{f}}{\alpha\int\left( \frac{\partial\xi_{LS}}{\partial c}\vec{b}\cdot\vec{n}ds \right)}, \text{where}$  $F_{P}=\int\Delta pds$  $F_{S}=\int\kappa\left( \xi_{LS}\left( \vec{x},t \right)-\xi_{LS}\left( \vec{x},0 \right) \right)\vec{b}\cdot\vec{n}ds$  $F_{m,V}=\alpha\int\frac{\partial^{2}\xi_{LS}}{\partial c^{2}}\left( \frac{dc}{dt} \right)^{2}\vec{b}\cdot\vec{n}ds$  $F_{f}=\alpha\int\Xi\left( \vec{x} \right)f_{\theta}\left( \vec{x} \right)\frac{\partial^{2}}{\partial t^{2}}\left( \frac{c\left( t \right)}{c_{max}}f_{ax}\left( \vec{x},t \right) \right)\vec{b}\cdot\vec{n}ds$ | (S |
| --- | --- | --- |

In the above equation, *F_P_*, *F_S_*, *F_m,V_* and *F_f_* represent forces due to pressure difference across the leaflet surface, restoring forces due to tissue elasticity, inertial effects arising from non-linear mapping functions and leaflet flutter, respectively. In this manner, the system is simplified from being comprised of numerous coupled partial differential equations to a single ODE per leaflet. The structural subsystem can thus be reduced from being comprised of $\mathcal{O}\left( {10}^{4} \right)$ coupled partial differential equations, in the context of finite element method (FEM)-based modeling, to a single ordinary differential equation per leaflet. S3 Fig (a) shows different leaflet kinematics which can be modeled using the proposed model. Different leaflet shapes can be obtained by tuning the mapping function $\xi_{i}\left( \vec{x},t \right)$, based on the type of bioprosthetic valve design. Moreover, modeled leaflet flutter is illustrated in the right panel, which shows an axially traveling wave in several snapshots of the fluttering leaflet centerline in the “fully-open” configuration. Different valvular pathologies and defects can also be easily modeled: for instance, individual leaflet stiffness/ mass can be increased to simulate aortic stenosis. Likewise, the valve can be comprised of two leaflets or have a fusion between two cusps, forming a type-0 or type-1 bicuspid aortic valve (BAV) phenotypes, respectively.


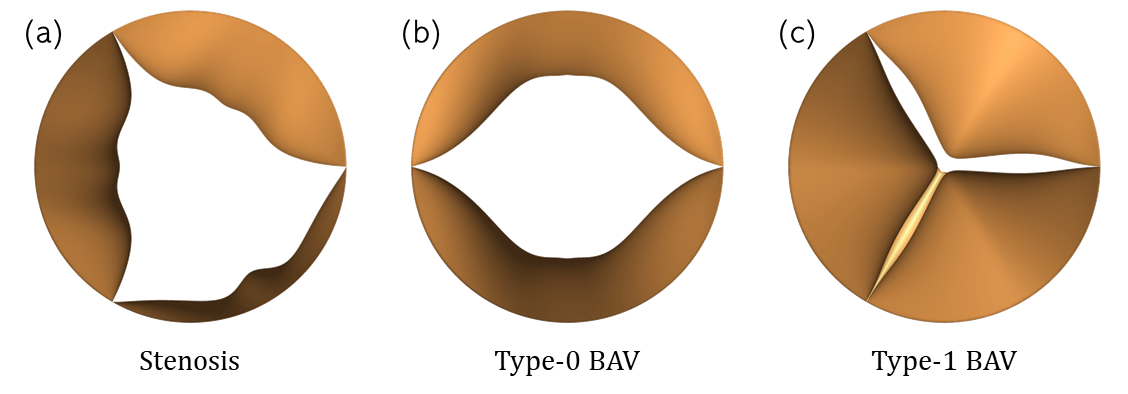


S3 Fig: Different valve pathologies which have been simulated using the rDOF valve model: (a) aortic stenosis, (b) type-0 and (c) type-1 bicuspid aortic valves.

An example of how model parameters can be tuned to describe observed leaflet kinematics is illustrated in S4 Fig. The top panel shows three high-speed imaging snapshots obtained from *in vitro* experiments of a Sorin Mitro Flow bioprosthetic AV (Sorin Group USA Inc., Arvada, CO) during its opening phase (image courtesy Dr. Lakshmi Prasad Dasi, Georgia Institute of Technology). The bottom panel shows corresponding *in silico* valve shapes obtained from simulations. It is observed that except for some fabrication defects such as delayed leaflet opening due to the rDOF model can replicate observed leaflet kinematics in BPVs reasonably well.


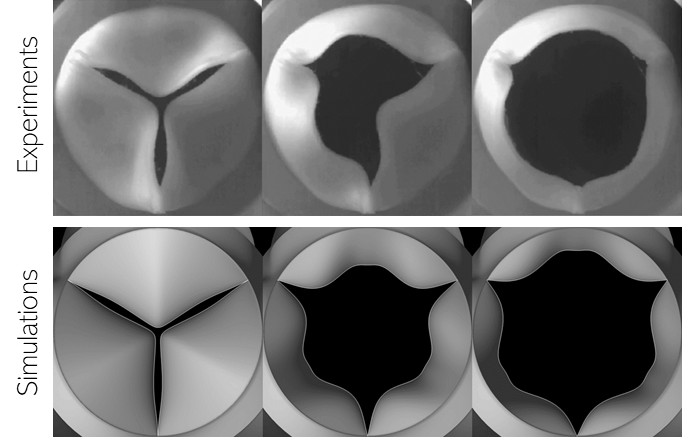


S4 Fig: Comparison of valve configuration at three instances during leaflet opening using (top) high-speed imaging of in vitro experiments using bioprosthetic valves and (bottom) present simulations.
