# Supplementary material for: Changes in aorta hemodynamics in Left-Right Type 1 bicuspid aortic valve patients after replacement with bioprosthetic valves: An in-silico study
Source: PLoS One. 2024 Apr 16;19(4):e0301350. doi: 10.1371/journal.pone.0301350 (PMC11020955; doi:10.1371/journal.pone.0301350)
Supplement: S2 Appendix — (DOCX) [file pone.0301350.s002.docx]

# SB Appendix: Adaptation of Valve Model to Patient-specific Anatomy

The circular section idealized AV model, described in the Appendix SA, suffers several limitations: the left ventricular outflow tract (LVOT) has been observed to have an elliptic cross-sectional shape, particularly in individuals with aortic stenosis^41^. Native and transcatheter valves normally conform to the non-circular annular shape and might exhibit cusp-wise differences in the leaflets, both of which affect the aortic jet shape and downstream hemodynamics. The idealized AV model used in conjunction with a canonical straight-tube aorta model does not distinguish between the non/ left/ right coronary cusps of the aortic sinus. In applications where flow in the sinus is of importance, such a simplified model might not give an accurate assessment of hemodynamic parameters in the valve vicinity. Fusion between leaflets or reduced mobility due to calcification affects the anatomic orifice area (AOA) of the valve, and consequently, aortic valve parameters such as jet velocity, tilting and transvalvular gradient. The canonical valve model would fall short in obtaining an accurate representation of these quantities.

To adapt the rDOF valve model to patient-specific anatomy, individual patient CT-scan data are incorporated in the model development process as follows: ascending aorta blood volume is segmented for individual patients using Materialise MIMICS (Materialise NV, Leuven, Belgium), and is converted to 3D triangular surface meshes using Materialise 3-Matic. The circular section valve model is axially positioned such that the valve skirt is aligned with the aortic annulus and the valve axis is oriented with the local direction of the aorta axis. The cross-sectional shape of the valve is then transformed to match that of the annulus, which is extracted as the intersection of a plane passing through the axis of the aorta at the annulus and oriented with the local tangent vector, as shown in S5 Fig (a). The resulting shape of the annulus is illustrated in S5 Fig (a) and is used to adjust the shape of the circular shaped canonical AV model. A generic point on the valve mesh with azimuthal coordinate $\theta$ in its fully open configuration is radially stretched (blue arrows) by a factor $f_{S}\left( \theta\right)= R_{S}\left( \theta\right)/R_{B}\left( \theta\right)$ where $R_{S}$ and $R_{B}$ represent its radial distances in the canonical and patient-fitted valve models, measured from their respective centroids, so that its new shape conforms to that of the aortic annulus. The local range-of-motion vector $\vec{b}$ is likewise stretched by the same factor $f_{S}\left( \theta\right)$, which is then used to compute the valve closed shape. The resulting transformed valve model for one patient included in the study is illustrated in S5 Fig (c).


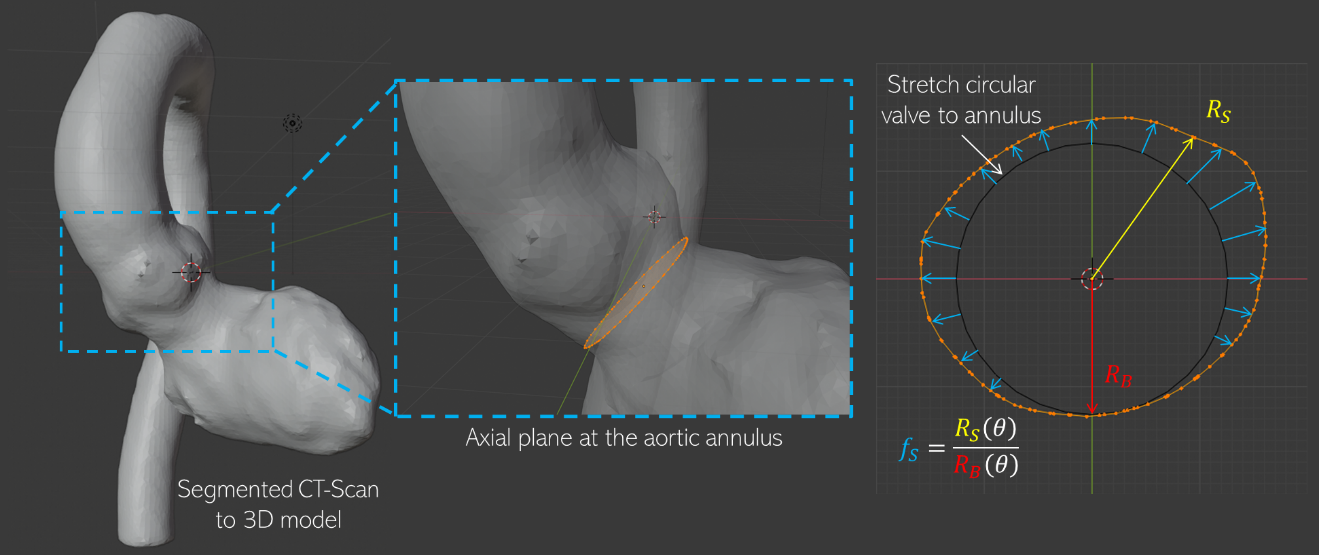


S5 Fig: Adaptation of canonical valve model to patient-specific annulus morphology: (a) identification of annular plane, (b) stretch transformation for a circular section to fit the annular shape and (c) new valve shape adapted to the patient-specific anatomy post transformation.

In this manner, the canonical valve model of circular cross-section described in Appendix SA is adapted to conform to the annular shape of each patient anatomy. It is evident that simple geometric transformations on canonical models can help accommodate large morphological variabilities associated with patient-specific modeling, thus expanding the ability of reduced order models to represent physiological conditions more accurately.
