# Supplementary material for: Changes in aorta hemodynamics in Left-Right Type 1 bicuspid aortic valve patients after replacement with bioprosthetic valves: An in-silico study
Source: PLoS One. 2024 Apr 16;19(4):e0301350. doi: 10.1371/journal.pone.0301350 (PMC11020955; doi:10.1371/journal.pone.0301350)
Supplement: S3 Appendix — (DOCX) [file pone.0301350.s003.docx]

# SC Appendix: Solution Verification

In this section, we describe results from a grid convergence study performed with Patient 4’s root and ascending aorta (S6 Fig (a)) anatomy with a left-right fused bicuspid valve. Three different grid resolutions were tested with minimum grid spacings of approximately 1.00 mm, 0.50 mm and 0.25 mm (S6 Fig (b)). The corresponding cases are labeled, based on the level of grid refinement, as ‘Coarse’, ‘Baseline’ and ‘Fine’, respectively. Each simulation is driven using a flow-rate profile shown in S6 Fig (c), such that the cardiac output (CO) is approximately 5 lpm. The annular area is A_0_ = 4.15 cm^2^, resulting in a peak LVOT velocity of approximately 106 cm/s and a peak Reynolds number $Re = \frac{U_{peak}}{\nu}\sqrt{\frac{4A_{0}}{\pi}}=6,135$. A homogenous Neumann pressure boundary condition is used at the inflow boundary (LVOT) and pressure in the aorta is computed relative to its value at the outflow boundary (aortic arch).


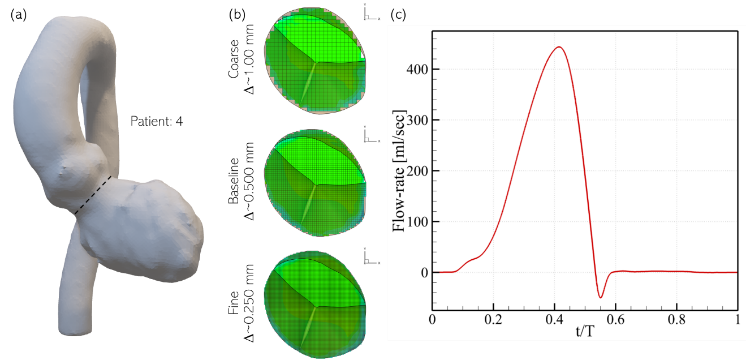


S6 Fig: (a) Left ventricle and aorta morphology for patient 4, (b) adapted bicuspid valve morphology showing the tested three grids in this convergence study, and (c) flow-rate profile used to drive the simulations.

To test whether the solution converges, we first compare integral quantities relating to valve motion and blood flow inside the aorta. S7 Fig shows comparison of the evolution of the PVOA and volume averaged flow kinetic energy in the three simulations: both plots demonstrate that the corresponding computed quantity approaches its respective fine grid solution as the grid resolution is increased. Specifically, the root-mean-squared (RMS) relative PVOA error in both cases was less than 5%. Likewise, the RMS relative error in flow kinetic energy decreased from 15.2% to 10.7%.


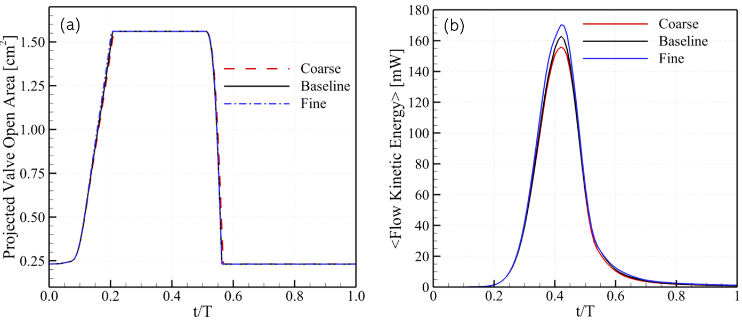


S7 Fig: Comparison of time-history of (a) projected valve open area (PVOA) and (b) volume-averaged flow kinetic energy computed in the coarse, baseline and fine grid simulations.

Next, we compare axial velocity contours, computed as $u_{N}=u\cdot n$, in four slices in the ascending aorta with local normal vector $n$, as shown in S8 Fig (a). We observe that prominent features of the aortic jet, identified by contours of positive axial velocity are well captured with each grid resolution in each analysis plane. For instance, the jet shape and magnitude as it emerges from the valve (slice 1), its anterior shift and its consequent distortion into a crescent shape in distal sections (slices 3 & 4) show similar distributions with each grid.


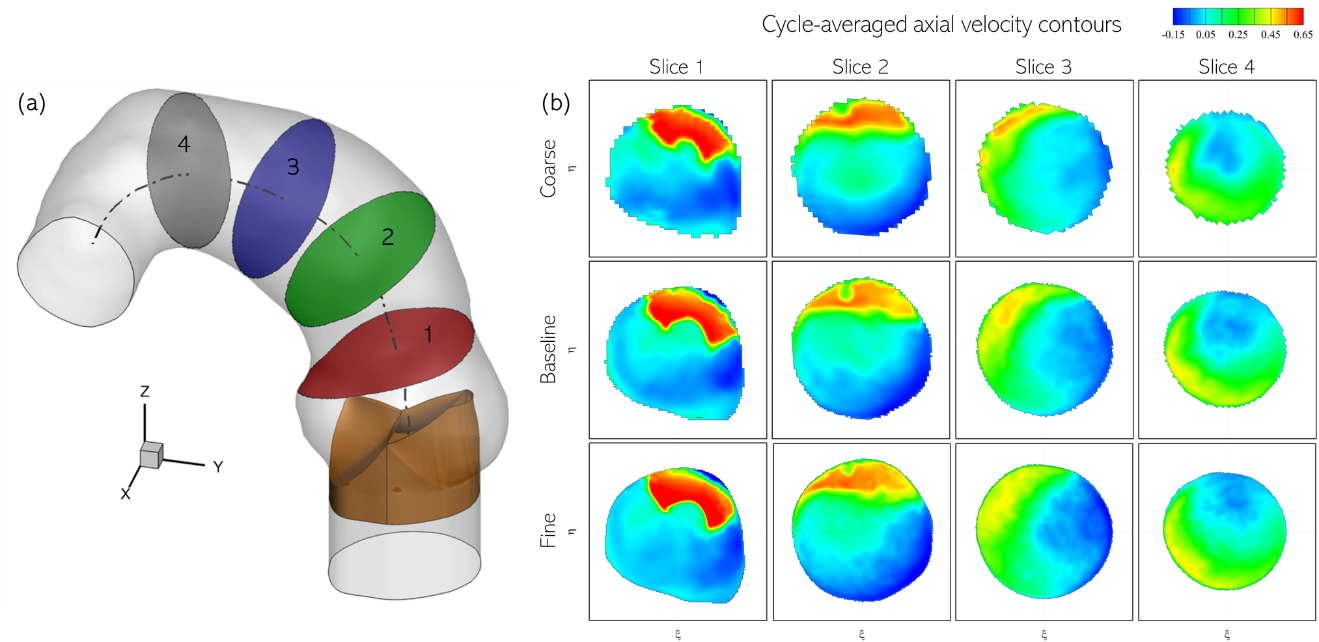


*S8 Fig: (a) Four axial analysis planes and (b) cycle-averaged axial velocity contours in each analysis plane using the three grids.*
