# Supplementary material for: Changes in aorta hemodynamics in Left-Right Type 1 bicuspid aortic valve patients after replacement with bioprosthetic valves: An in-silico study
Source: PLoS One. 2024 Apr 16;19(4):e0301350. doi: 10.1371/journal.pone.0301350 (PMC11020955; doi:10.1371/journal.pone.0301350)
Supplement: S4 Appendix — (DOCX) [file pone.0301350.s004.docx]

# SD Appendix: Model Validation

Hemodynamic measurements from the de-identified pre-intervention transthoracic echo (TTE) report for Patient 1 are presented in S1 Table. The 3D model segmented from pre-intervention CT-scan was scaled so that that the LVOT cross-section area was matched with that calculated using the LVOT diameter ($2.1$ cm) from the TTE report and assuming a circular cross-section.

S1 Table: Relevant information from Patient 1's pre-intervention TTE, which is used to design and validate the rDOF valve model.

| **Input/Output** | **Parameter** | **Measured Value** | **Input/Output (% Error)** |
| --- | --- | --- | --- |
| Input | LVOT Diam | 1.1 cm | 1.1 cm |
|  | **AV Doppler** |  |  |
| Input | LVOT V_max_ | 0.98 m/s | 0.98 m/s |
| Input | LVOT V_mean_ | 0.72 m/s | 0.72 m/s |
| Input | LVOT maxPG | 3.86 mmHg | - |
| Input | LVOT meanPG | 2.30 mmHg | - |
| Input | LVOT VTI | 27.1 cm | 27.6 cm (+1.84%) |
| Output | AV V_max_ | 5.32 m/s | 5.21 m/s (-2.06%) |
| Output | AV maxPG | 113.20 mmHg | 108.57 mmHg (-4.09%) |

In the absence of clinically measured time varying LVOT flow profile obtained using continuous wave (CW) Doppler echo, an alternate velocity profile is synthesized using the hyperbolic tangents as shown in equation (S8). The profile is characterized using the following parameters:

1. peak velocity, $W$, which represents the maximum velocity attained by the profile.
2. start and end times denoted by $t_{S}$ and $t_{E}$, respectively, which represent the times at which the velocity profile reaches 50% of the peak value during the acceleration and deceleration phases.
3. rise and fall times, denoted by $t_{R}$ and $t_{F}$, respectively, which represent the rates of velocity change during the acceleration and deceleration phases.

The peak velocity is set to the measured LVOT $V_{max}$ (=0.98 m/sec), the start time ($t_{S}$) 0.25 and a heart rate (HR) of 60 bpm was assumed. The values for $t_{E}$, $t_{R}$ and $t_{F}$ are chosen via an optimization process such that the velocity-time integral (VTI) of the resulting profile $\left( 27.6\text{ cm} \right)$ closely matches the measured LVOT VTI $\left( 27.1\text{ cm} \right)$. This ensures that while the exact shape of the velocity profile is unknown, the peak and average velocities represented by the synthesized profile match the respective measured values. The resulting optimized velocity profile is illustrated in S9 Fig (a).

|  | $w\left( \hat{t} \right)=W\left[ \tanh\left( \frac{\hat{t}-t_{S}}{t_{R}} \right)-\tanh\left( \frac{\hat{t}-t_{E}}{t_{F}} \right) \right]$ | ( |
| --- | --- | --- |


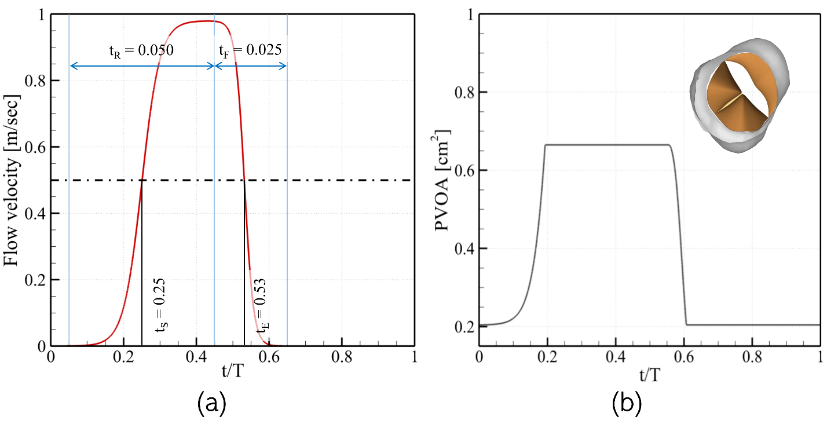


S9 Fig: (a) Optimized flow rate profile used for model validation tuned to fit TTE parameters and (b) time-varying PVOA for Patient 1's AV subject to the optimized flow-rate profile.

In the simulation, maximum $V_{AV}$ was computed as the axial component of the peak systolic velocity vector, averaged over the cross-section of the aortic jet at the *vena contracta* (S10 Fig (a)) before turbulent jet separation occurs. The jet is visualized in Figure 10 (b) as the velocity normal to the *vena contracta* plane and it has a crescent shape typically observed with a type-1 BAV, resulting due to the singular mobile leaflet. The maximum $V_{AV}$ from the simulated case was 5.21 m/sec which is $2.06\%$ lower than the corresponding observed value of 5.32 m/sec. The corresponding peak gradient across the valve from the simulation was 108.57 mmHg, a $-4.09\%$ deviation from the corresponding reported value of 113.20 mmHg. These results are summarized in S2 Table. Thus, the results presented in Appendices SA-SD demonstrate the versatility of the rDOF valve model and its ability to accurately model blood flow in canonical and individualized anatomy.


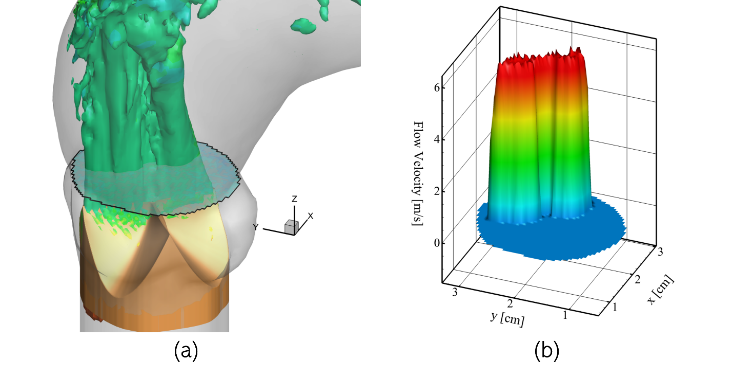


*S10 Fig: (a) Isosurfaces of velocity magnitude indicating peak systolic jet shape and an axial slice at the vena contracta (VC), (b) aortic jet shape determined using contours of velocity magnitude at the VC.*

S2 Table: Comparison of bicuspid valve performance in Patient 1 as observed using echocardiography and predicted using simulations.

| Peak Anatomic Orifice Area [cm^2^] | | Maximum AV velocity [m/s] | | Peak gradient [mm Hg] | |
| --- | --- | --- | --- | --- | --- |
| Echo + Continuity | Simulation (Error) | Echocardiography | Simulation (Error) | Echocardiography | Simulation (Error) |
| 0.638 | 0.66 (+3.44 %) | 5.32 | 5.21 (-2.06 %) | 113.20 | 108.57 (-4.09 %) |
